# Transfection of microRNA Mimics Should Be Used with Caution

**DOI:** 10.3389/fgene.2015.00340

**Published:** 2015-12-02

**Authors:** Hyun Yong Jin, Alicia Gonzalez-Martin, Ana V. Miletic, Maoyi Lai, Sarah Knight, Mohsen Sabouri-Ghomi, Steven R. Head, Matthew S. Macauley, Robert C. Rickert, Changchun Xiao

**Affiliations:** ^1^Department of Immunology and Microbial Science, The Scripps Research InstituteLa Jolla, CA, USA; ^2^Kellogg School of Science and Technology, The Scripps Research InstituteLa Jolla, CA, USA; ^3^Program on Immunity and Pathogenesis, Sanford-Burnham Medical Research InstituteLa Jolla, CA, USA; ^4^Department of Cell and Molecular Biology, The Scripps Research InstituteLa Jolla, CA, USA; ^5^Department of Chemical Physiology, The Scripps Research InstituteLa Jolla, CA, USA; ^6^Next Generation Sequencing Core, The Scripps Research InstituteLa Jolla, CA, USA

**Keywords:** transient transfection, microRNA mimics, miR-17~92, miR-155, high molecular weight RNA species, guide strand mutation, unnatural passenger strand

## Abstract

Transient transfection of chemically synthesized microRNA (miRNA) mimics is being used extensively to study the functions and mechanisms of endogenous miRNAs. However, it remains unclear whether transfected miRNAs behave similarly to endogenous miRNAs. Here we show that transient transfection of miRNA mimics into HeLa cells by a commonly used method led to the accumulation of high molecular weight RNA species and a few hundred fold increase in mature miRNA levels. In contrast, expression of the same miRNAs through lentiviral infection or plasmid transfection of HeLa cells, transgenic expression in primary lymphocytes, and endogenous overexpression in lymphoma and leukemia cell lines did not lead to the appearance of high molecular weight RNA species. The increase of mature miRNA levels in these cells was below 10-fold, which was sufficient to suppress target gene expression and to drive lymphoma development in mice. Moreover, transient transfection of miRNA mimics at high concentrations caused non-specific alterations in gene expression, while at low concentrations achieved expression levels comparable to other methods but failed to efficiently suppress target gene expression. Small RNA deep sequencing analysis revealed that the guide strands of miRNA mimics were frequently mutated, while unnatural passenger strands of some miRNA mimics accumulated to high levels. The high molecular weight RNA species were a heterogeneous mixture of several classes of RNA species generated by concatemerization, 5′- and 3′-end tailing of miRNA mimics. We speculate that the supraphysiological levels of mature miRNAs and these artifactual RNA species led to non-specific changes in gene expression. Our results have important implications for the design and interpretation of experiments primarily employing transient transfection of miRNA mimics.

## Introduction

MicroRNAs (miRNAs) are endogenously encoded single stranded RNAs of about 22 nucleotides (nts) in length that play essential roles in a large variety of physiological processes (Ambros, 2004; Bushati and Cohen, 2007; Krol et al., 2010; Palanichamy and Rao, 2014). During their biogenesis, miRNA genes produce nascent transcripts with stem-loop structures (pri-miRNAs), which are processed sequentially by the Drosha-DGCR8 complex and Dicer to yield mature miRNA duplexes (miRNA/miRNA^*^) (Kim et al., 2009). These miRNA duplexes are subsequently integrated into RNA-induced silencing complexes (RISC), whose core component is one of the Argonaute family proteins (AGO1-4) in mammalian cells. Once a miRNA duplex is loaded, RISC quickly removes the passenger strand (miRNA^*^) either by cleavage or by an unwinding and release process (Ha and Kim, 2014). The guide strand directs RISC to target mRNAs, which are recognized through partial sequence complementarity *via* the seed sequence located at nucleotide positions 2–8 of the mature miRNA. The functional consequences of miRNA-target mRNA interactions can be translation repression, mRNA degradation, or both (Fabian et al., 2010; Wilczynska and Bushell, 2015). The molecular mechanisms underlying these two distinct functional consequences have been under extensive investigation but remain unresolved (Jin and Xiao, 2015; Jonas and Izaurralde, 2015).

MiRNA mimics are chemically synthesized double-stranded RNA molecules imitating mature miRNA duplexes. Chemical modifications not present in endogenous miRNAs (Wang, 2011; Thomson et al., 2013), as well as nucleotide changes in the passenger strands (Lim et al., 2005; Garcia et al., 2011), are often introduced to miRNA mimics to improve their stability, to facilitate guide miRNA loading to RISC, and to selectively exclude the passenger strand. Delivery of miRNA mimics into cells can bypass the endogenous miRNA biogenesis pathway and alter miRNA abundance instantly. Transient transfection can efficiently deliver miRNA mimics into *in vitro* cultured mammalian cells, and has been taken for granted as a fast, easy, and economical way to gain insights into the functions and mechanisms of action of endogenous miRNAs. However, the proprietary chemical modifications and formulations of miRNA mimics are often not disclosed to users, thereby increasing the chance of performing misleading experiments (Git, 2012). Also, the mechanisms of action of chemically synthesized miRNA mimics presumably recapitulate that of endogenous miRNAs, but supporting evidence is quite limited despite their widespread use. Thus, a recent study employing this approach led to the conclusion that miRNAs predominantly act to decrease target mRNA levels rather than decreasing translation efficiency (Guo et al., 2010). By contrast, analyses of select sets of functionally relevant target genes in mice with loss- and gain-of function mutations for individual miRNA genes often showed significant changes in protein concentrations, but with marginal or no alterations in mRNA levels (Zhao et al., 2005, 2007; Lu et al., 2007, 2009; Vigorito et al., 2007; Van Rooij et al., 2007; Dorsett et al., 2008; Boettger et al., 2009; Callis et al., 2009; O'connell et al., 2009, 2010; Williams et al., 2009; Biton et al., 2011; Boldin et al., 2011; Liu et al., 2011; Ma et al., 2011; Sanuki et al., 2011; Shibata et al., 2011; Bian et al., 2013; Danielson et al., 2013; Hasuwa et al., 2013; Henao-Mejia et al., 2013; Stadthagen et al., 2013; Wang et al., 2013, 2015; Agudo et al., 2014), corroborating the initial findings in the field that miRNAs repress the protein output of target genes without significantly effecting their mRNA levels in animals (Lee et al., 1993; Wightman et al., 1993).

We speculated that the discrepancy between these two types of studies regarding the predominant mechanism of miRNA action stems from the transient transfection approach, which may not recapitulate the actions of endogenous miRNAs under physiological conditions (Jin and Xiao, 2015). To address this issue, we performed transient transfection of mimics of several oncogenic miRNAs into HeLa cells following commonly used experimental conditions, and examined their expression levels over time by Northern blot. In addition, we compared the effect of transient transfection of miRNA mimics on target gene mRNA and protein levels with the effect of lentiviral expression, plasmid transfection and transgenic expression of the same miRNAs. We also performed small RNA deep sequencing analysis of cells transiently transfected with miRNA mimics to determine the exact sequences of RNA species arising from these mimics. Our results have profound implications for the design of miRNA experiments and for the interpretation of experimental results from studies predominantly relying on the transient transfection approach.

## Materials and methods

### Mice and ethics statement

The generation of miR-17~92 Tg (Jax stock 008517), CD19-Cre (CD19^cre∕+^) (Jax stock 006785), and miR-155 transgenic mice was previously reported (Rickert et al., 1997; Costinean et al., 2006; Xiao et al., 2008). MiR-17~92 Tg mice were crossed with CD19-Cre mice to generate miR-17~92 Tg/Tg; CD19-Cre (TG) mice (Jin et al., 2013). CD19-Cre mice were used as control. All animal experiments were approved by Animal Care and Use Committee of The Scripps Research Institute.

### Purification of B cells and *in vitro* stimulation

Spleen and peripheral lymph nodes were collected from 8-week-old mice of indicated genotypes. Primary B cells were purified by depleting cells positive for AA4.1 (CD93, eBioscience- 13-5892-85), CD43 (DB Pharmingen- 553269), or CD5 (BioLegend-100604) using MACS LD columns (Miltenyi Biotec) following manufacturer's instructions. The purified B cells were cultured at 5 × 10^6^ cells/ml, and rested for 3 h before harvesting (naïve) or activated for indicated amounts of time with 2 μg/ml anti-IgM (for miR-17~92 transgenic B cells) or 25 μg/ml LPS and 5 ng/ml IL-4 (for miR-155 transgenic B cells) in 37°C cell culture chambers. At the time of harvesting, live cells were purified using Ficoll following manufacturer's instructions (GE Healthcare, Ficoll Paque PLUS, 17-1440-02), achieving >90% viability and >98% B cell purity (B220^+^ CD19^+^).

### Cell culture

HeLa cells were cultured in DMEM with glutamax (Dulbeccos's modified eagle medium with glutamax, Gibco 10569) supplemented with 10% fetal bovine serum and 1% of 100 units/ml penicillin/streptomycin (100x stock). The OCI-Ly3, OCI-Ly1, and OCI-Ly19 cell lines were obtained from Dr. Minden (University of Toronto) and cultured in Iscove's Modified Dulbecco's Medium (Life Technologies) containing 20% Fetal Bovine Serum (FBS; HyClone), and 100 units/ml penicillin and 100 μg/ml streptomycin (Gibco). The BJAB, Ramos, Raji, SU-DHL-4 cell lines were obtained from the American Type Culture Collection (ATCC) and cultured in RPMI 1640 Medium containing 2 mM L-Glutamine (Gibco), 10% FBS, and 100 units/ml penicillin and 100 μg/ml streptomycin. All human lymphoma cell lines were cultured to approximately 1 × 10^6^ cell/ml at 37°C with 5% CO_2_ in T-175 flasks. Cells were removed, centrifuged (7 min, 270 rcf), and resuspended in PBS buffer (Corning Cellgro). Cells were counted, and 1.8 × 10^7^ cells were centrifuged. Following removal of the supernatant, cell pellets were frozen at −80°C until further processing for Northern blot.

### Transient transfection of miRNA mimics

5 × 10^5^ HeLa cells were seeded in 60 mm plates 18–24 h prior to transfection. Media were replaced with antibiotics-free media 6–12 h before transfection. Synthetic miRNA mimics (from Dharmacon or Shanghai GenePharma) were transfected into cells following the Dharmafect1 transfection protocol (T-2001-03). Twenty-four hours later, cells were split into two 60 mm plates in antibiotics-containing media and cultured for an additional 48 h. Cells were then washed twice with PBS and lysed in TRIzol reagent (Life Technology, #15596) or in 1% NP-40 lysis buffer containing 1% Nonidet P-40, 150 mM NaCl, 50 mM Tris-Cl (pH 8.0), 1 mM sodium orthovanadate, 1 mM DTT and proteinase inhibitors (halt inhibitor, Thermo #78442) for total RNA and protein extraction, respectively.

### Synthetic miRNA mimics

The following synthetic miRNA mimics were used in this study:

Mimic Transfection Control with Dy547 (cel-mir-67 conjugated with Dy547), Dharmacon CP-004500-01-10
miRIDIAN microRNA Mimic Negative Control #1 (cel-mir-67), Dharmacon CN-001000-01-10
miRIDIAN Mimic hsa-miR-17, Dharmacon C-300485-05-0005
miRIDIAN Mimic hsa-miR-18a, Dharmacon C-300487-05-0005
miRIDIAN Mimic hsa-miR-19a, Dharmacon C-300488-03-0005
miRIDIAN Mimic hsa-miR-20a, Dharmacon C-300491-03-0005
miRIDIAN Mimic hsa-miR-19b, Dharmacon C-300489-03-0005
hsa-miR-92a, custom synthesized by Shanghai GenePharma
miRIDIAN Mimic hsa-miR-155, Dharmacon C-300647-05-0010

### Stable cell line generation

Generation of miR-17~92-expressing lentivirus was previously described (Hong et al., 2010). HeLa cells were seeded in 48-well plates at a density of 5 × 10^3^ cells/well on the afternoon prior to transduction with control or miR-17-92-expressing lentiviruses (LV-Control and LV-miR-17~92, respectively). On the afternoon of the following day, different amounts of LV-Control or LV-miR-17~92 lentiviruses corresponding to multiplicities of infection (MOI) 10, 20, 50, and 100 were added to cells and overnight infection was performed in the presence of 8 μg/ml polybrene. Media was replaced 16 h after adding viruses. Transduction efficiency was monitored by flow cytometry analysis of GFP, which is encoded by lentiviruses. Transduced cells were further expanded to generate low-passage stocks of stable cell lines.

### Plasmid transfection

Transfection of miR-17~92-expresing plasmid was previously described (Xiao et al., 2008). Briefly, 2 × 10^5^ HeLa cells were plated into 12 well plates 24 h before transfection. At 70% confluency, 0.4 μg of miR-17~92-expressing pCNX2 plasmid (Plasmid-miR-17~92) or empty pCNX2 plasmid (Plasmid-Empty) were transfected into each well using FugeneHD (Promega #E2311) following manufacturer's instructions. The miR-17~92-expressing-lentivirus (LV-miR-17~92), plasmid (Plasmid-miR-17~92), and transgene (TG) used in this study all contain the same insert, a 1007 bp fragment of human genomic DNA encoding miR-17~92 (human chr13: 91350568–91351574) (Xiao et al., 2008).

### Northern blot

MiRNA Northern blot was performed as described previously (Xiao et al., 2007, 2008). First, 20 μg TRIzol-extracted total RNA in less than 10 μl RNase-free water was mixed with 20 μl Gel Loading Buffer II (Ambion, #8547). This loading buffer contains 95% formamide, 18 mM EDTA and 0.025% SDS, therefore efficiently denatures potential RNA duplexes. The samples were further denatured at 95°C for 5 min and immediately cooled down on ice to prevent re-annealing. The denatured RNA samples were separated on 10% denaturing polyacrylamide gels. The gel was made as the following: 16.8 g urea, 4 ml of 5x TBE solution (27 g Tris Base, 13.7 g boric acid, 10 ml of 0.5 M EDTA pH 8.0, fill up DEPC water to 500 ml), and 16 ml DEPC water were mixed and heated to 70°C for 20 min to dissolve urea completely. The solution was cooled down at room temperature. Then 10 ml 40% (w/v) Acrylamide/Bis-Acrylamide 37.5:1 solution (Bioworld, 40120040-3) was added to make a total of 40 ml solution. Finally, 240 μl of 10% APS and 32 μl TEMED were added and mixed before pouring into RNase-free gel cassettes (18 cm × 16 cm). Once the gel was solidified, the samples were separated in 0.5x TBE buffer with constant power (5 W) until the bromophenol blue dye from the Gel Loading Buffer II reached about 4 cm to the end of the gel. The gel was electrotransfered to a positively charged nylon membrane (GE Healthcare, Amersham Hybond-N+, RPN2020b) for 8–10 h in a cold room at constant voltage (30 V) in 0.5x TBE buffer with stirring. The transferred membranes were briefly washed with 0.5x TBE buffer, dried on Whatman paper for 10 min, and UV-cross-linked to fix the transferred RNAs to the membrane. For miRNA detection, DNA oligonucleotides antisense to mature miRNAs were purchased from Integrated DNA Technologies (Coralville, IA) and used as probes. The synthesized DNA oligos were 5′-end labeled using γ-^32^P ATP (Perkin Elmer, BLU002A100UC) with T4 Polynucleotide Kinase (NEB, M0201S) following manufacturer's instructions. The labeled oligos were purified using MicroSpin G25 column (GE Healthcare, 27-5325-01). miRNA signals were detected with probes for individual miRNAs, or a mixture of probes detecting all members of a miRNA subfamily (i.e., miR-17 subfamily). For example, the probe mixture for the miR-17 subfamily contains probes for miR-17, miR-20a, miR-106a, miR-20b, miR-106b, and miR-93, the probe mixture for the miR-18 subfamily contains probes for miR-18a and miR-18b, the probe mixture for the miR-19 subfamily contains probes for miR-19a and miR-19b, and the probe mixture for the miR-92 subfamily contains probes for miR-92, miR-363, and miR-25. U6 snRNA was used as an internal control for normalization. The purified probes were pre-heated in hybridization buffer (40 ml of 5 M NaCl, 10 ml of 1 M Tris, pH 7.5, 20 g of Dextran sulfate sodium salt, 10 ml of 20% (w/v) SDS, 5 ml of sonicated salmon sperm DNA (10 mg/ml), and 135 ml water with a final volume of 200 ml) at 65°C. The membranes were pre-hybridized with the hybridization buffer for 2–3 h and hybridized with pre-heated probes at 42°C overnight. The hybridized membranes were washed with Buffer I (2x SSC, 0.1% SDS) and Buffer II (1x SSC, 0.1% SDS) at room temperature for 30 min each. Additional washing with Buffer III (0.5x SSC, 0.1% SDS) was included when the background signals were high. The membranes were exposed to a phospho-screen, which was subsequently scanned on a STORM 860 phosphorimager. Data were analyzed using the ImageQuant software.

**Table:** 

**miRNA subfamily**	**Mature miRNAs**	**Northern blot probes (seed sequences are underlined)**
miR-17 subfamily:	miR-17-5p	ACT ACC TGC ACT GTA A*GC ACT TT*G
	miR-20a	CTA CCT GCA CTA TAA *GCA CTT T*A
	miR-106a	CTA CCT GCA CTG TTA *GCA CTT T*G
	miR-20b	CTA CCT GCA CTA TGA *GCA CTT T*G
	miR-106b	ATC TGC ACT GTC A*GC ACT TT*A
	miR-93	CTA CCT GCA CGA ACA *GCA CTT T*G
miR-18 subfamily:	miR-18a	CTA TCT GCA CTA GAT *GCA CCT T*A
	miR-18b	CTA ACA GCA CTA GAT *GCA CCT T*A
miR-19 subfamily:	miR-19a	TCA GTT TTG CAT AGA *TTT GCA C*A
	miR-19b	TCA GTT TTG CAT GGA *TTT GCA C*A
miR-92 subfamily:	miR-92	CAG GCC GGG ACA A*GT GCA AT*A
	miR-363	TAC AGA TGG ATA CC*G TGC AAT* T
	miR-25	TCA GAC CGA GAC AA*G TGC AAT* G
miR-155	miR-155	CCC CTA TCA CAA TT*A GCA TTA* A
Antisense miR-17	n.a	AAC AAA GTG CTT ACA GTG
cel-mir-67	cel-mir-67	TCT ACT CTT TCT AGG A*GG TTG TG*A
U6 snRNA (control)	n.a	TAT GTG CTG CCG AAG CGA GCA C

### Western blot

HeLa cells were lysed in 1% NP-40 lysis buffer containing 1% Nonidet P-40, 150 mM NaCl, 50 mM Tris-Cl (pH 8.0), 1 mM sodium orthovanadate, 1 mM DTT and proteinase inhibitors (halt inhibitor, Thermo #78442), and subjected to separation on 8 or 10% SDS-PAGE gels and Western blot (10 μg whole cell lysate per lane). Antibodies used for Western blot were anti-Bim (Cell Signaling, 2933), anti-Pten (Cell Signaling, 9559), anti-Phlpp2 (Bethyl, A300-661A-1), and anti-Ship1 (Cell Signaling, 2728).

### Real-time PCR

Total RNA was prepared and extracted as described above. cDNA libraries were generated using 1 μg total RNA with an iScript cDNA Syntehesis Kit (Bio-Rad, 170-8891), whose premix contains both random hexamer and oligo-dT primers. The subsequent quantitative PCR analysis was performed with Maxima SYBR Green/Rox Master Mix (Thermo, K0223) and gene specific primers. The reaction mixture was initially denatured for 10 min at 95°C, followed by 40 PCR cycles. Each PCR cycle consists of a denaturing step (95°C for 15 s) and a primer annealing/extension step (60°C for 60 s). The primers used in this study are:

human Actb(F): 5′-ACCTTCTACAATGAGCTGCG-3′
human Actb(R): 5′-CCTGGATAGCAACGTACATGG-3′
human Phlpp2(F): 5′-TGGAACACAAGACACTGGAC-3′
human Phlpp2(R): 5′-AGCTTATTTCTCTGCCCTGC-3′
human Pten(F): 5′-AAGGGACGAACTGGTGTAATG-3′
human Pten(R): 5′-GCCTCTGACTGGGAATAGTTAC-3′
human Bim(F): 5′-TCGGACTGAGAAACGCAAG-3′
human Bim(R): 5′-CTCGGTCACACTCAGAACTTAC-3′
human Isg15(F): 5′-ACTCATCTTTGCCAGTACAGG-3′
human Isg15(R): 5′-CAGCTCTGACACCGACATG-3′
human Oas1(F): 5′-CATCTGTGGGTTCCTGAAGG-3′
human Oas1(R): 5′-GAGAGGACTGAGGAAGACAAC-3′
human Ifna2(F): 5′-CCCATTTCAACCAGTCTAGCAG-3′
human Ifna2(R): 5′-TGTGGGTTTGAGGCAGATC-3′
human Ifnb1(F): 5′-AGGATTCTGCATTACCTGAAGG-3′
human Ifnb1(R): 5′-GGCTAGGAGATCTTCAGTTTCG-3′
human Mx1(F): 5′-GAAGATAAGTGGAGAGGCAAGG-3′
human Mx1(R): 5′-CTCCAGGGTGATTAGCTCATG-3′
human Eif2ak2(F): 5′-CGATACATGAGCCCAGAACAG-3′
human Eif2ak2(R): 5′-AGAATTAGCCCCAAAGCGTAG-3′
human Tlr3(F): 5′-TCAACTTTCTGATAAAACCTTTGCC-3′
human Tlr3(R): 5′-AGATGACAAGCCATTATGAGACA-3′
human Cxcl10(F): 5′-CCTTATCTTTCTGACTCTAAGTGGC-3′
human Cxcl10(R): 5′-ACGTGGACAAAATTGGCTTG-3′
human Irf7(F): 5′-TCCCCACGCTATACCATCTAC-3′
human Irf7(R): 5′-GAAGACACACCCTCACGC-3′
murine Actb(F): 5′-CTAAGGCCAACCGTGAAA G-3′
murine Actb(R): 5′-ACCAGAGGCATACAGGGACA-3′
murine Phlpp2(F): 5′-ATCTGGTCAGGAGACTGG A-3′
murine Phlpp2(R): 5′-GGATTCGATCCAGATGATCCA-3′
murine Pten(F): 5′-TGGGGAAGTAAGGACCAGAG-3′
murine Pten(R): 5′-GGCAGACCACAAACTGAGGA-3′
murine Bim (F):5′-gagatacggattgcacaggag-3′
murine Bim (R): 5′-cggaagataaagcgtaacagttg-3′

Differential expression was calculated according to the ΔΔCT method.

### Small RNA deep sequencing

#### Sample preparation

1 μg total RNA from non-transfected HeLa cells and HeLa cells transfected with an equal molar mixture of miR-17, 18a, 19a, 20a, 19b, and 92a (16.7 nM each with a total transfection concentration of 100 nM) was used for Next Generation Sequencing following the standard Illumina protocol, TruSeq_SmallRNA_SamplePrep_Guide_15004197_C.pdf, using 12 cycles of PCR.

#### 3′ adapter ligation

10 pmol of 3′-adenylated DNA adapter (5′-AppT GGAATTCTCGGGTGCCAAGGddC) is combined with 1 μg total RNA in a 4 μl volume, heated to 70°C for 2 min and then placed on ice. In a separate tube, 2 μl 10X T4 RNA Ligase 2 Truncated reaction buffer (NEB), 2 μl 50% PEG8000 (NEB), 1 μl RNase OUT (Lifetech) and 1 μl T4 RNA Ligase 2 Truncated enzyme (NEB) were combined and added to the RNA/3′adapter mix and incubated at 28°C for 1 h. This mixture was then heated to 70°C for 2 min.

#### 5′ adapter ligation

1 μl water, 1 μl 10 mM ATP, 1 μl T4 RNA Ligase and 1 μl 5′ RNA adapter (25 pmol, 5′-GUUCA GAGUUCUACAGUCCGACGAUC) were added and incubated at 28°C for 1 h.

#### Reverse transcription

1 μl of a 10 μM solution of RT primer (5′-GCCTTGGCACCCGAGAA TTCCA) was added to 6 μl of the 5′ adapter ligation mix above, heated to 70°C for 2 min and then transferred to ice. This was then combined with a solution containing 2 μl of 5x First Strand Buffer (Superscript II buffer, Lifetech), 0.5 μl of 12.5 mM dNTPs, 1 μl 0.1 M DTT, 1 μl RNaseOUT (Lifetech), and 1 μl Superscript II (200 U/μl). The reaction was incubated at 50°C for 1 h and then placed on ice.

#### cDNA library synthesis

The 12.5 μl reverse transcription reaction above was added to a tube containing: 30 pmol of Illumina Truseq Universal PCR primer (5′-AATGAT ACGGCGACCACCGAGATCTACACGTTCAGAGTTCTACAGTCCGA); 30 pmol of Illumina Truseq Barcoded PCR primer (5′-CAAGCAGAAG ACGGCATACGAGATXXXXXXGTGACTGGAGTTCCTTGGCACCCGAGAATTCCA, in which XXXXXX corresponds to standard Illumina Truseq barcodes); and 25 μl of 2x KAPA HiFi HotStart ReadyMix PCR Kit in a total volume of 50 μl. The reaction was heated to 98°C for 1 min, and then cycled 12 times with 98°C for 10 s, 60°C for 30 s, and 72°C for 15 s, followed by a single incubation at 72°C for 5 min. The resulting PCR products were isolated using AMPure XP PCR purification system (Agencourt, Beckman-Coulter), separated on a 4% Agarose gel, with PCR products cut in the 140–195 bps range, which corresponds to insert size of 20–75 bps. Library was extracted from the gel using Zymo agarose dissolve buffer (ADB) as specified by the Zymo manual and then run over a Zymo DNA Clean & Concentrator-25 column to purify. The prepared library was then loaded onto an Illumina NextSeq500 flowcell, sequenced for 75 bases of the insert and 7 bases of the index read using standard NextSeq500 sequencing reagents. Reads were then processed using CASAVA 1.8 and demultiplexed based on index sequences.

#### Data processing and bioinformatic analysis

Raw reads were preprocessed to remove the adaptors using cutadapt (Martin, 2011). Reads shorter than 15 nucleotides or longer than 80 nucleotides were discarded. Next, reads were aligned to the pre-built Bowtie indexed human genome, H. Sapiens UCSC hg19, using Bowtie short read aligner (http://bowtie-bio.sourceforge.net/index.shtml), and rRNA species were removed. The remaining non-rRNA reads were examined for quality control using FastQC software (http://www.bioinformatics.babraham.ac.uk/projects/fastqc/). The highly enriched reads (greater than 0.1% of total reads) were selected for further analysis. For each sample, known miRNAs were identified and enumerated using miRDeep2 software package (Friedländer et al., 2008). To analyze high molecular weight RNA species, reads between 25 and 74 nt were pre-selected and analyzed by ShortRead and Biostrings library from Bioconductor in R (Morgan et al., 2009). To isolate RNA species containing a string of mature miR-17~92 or mutated miR-17~92 sequences, vcountPattern function with max.mismatch = 5, with.indels = T option was used. The length distribution plot was generated using custom scripts in R. miRNA sequencing results are available in the NCBI Gene Expression Omnibus (GEO) database under the series accession identifier GSE73099.

## Results

### Dose–dependent effect of miRNA mimics on cell survival

**Figure 1:**
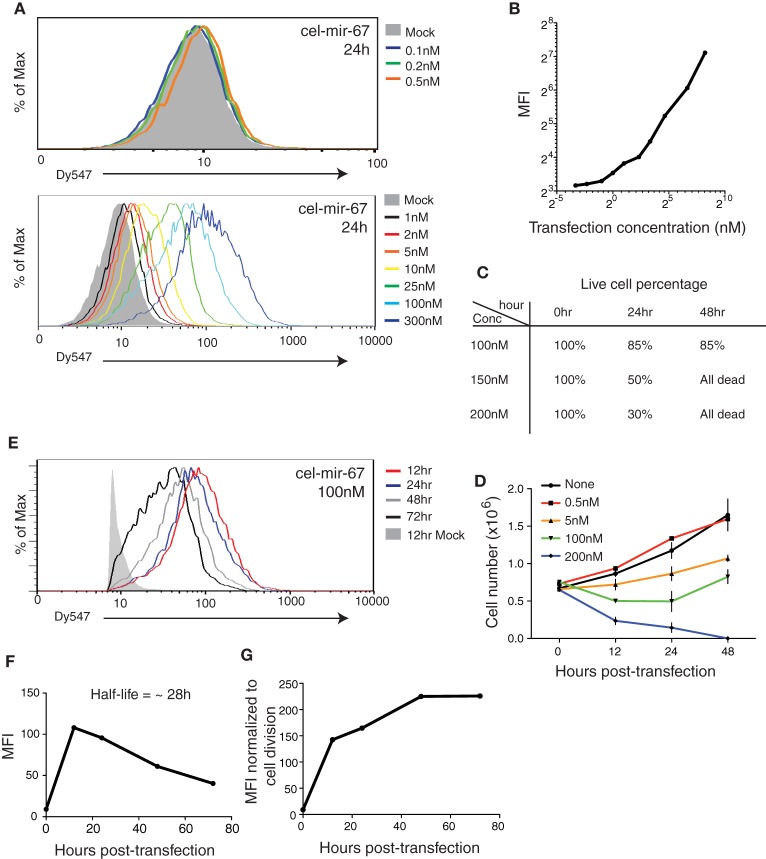
**Transient transfection of cel-mir-67 into HeLa cells. (A)** HeLa cells were transfected with indicated concentrations of Dy547-conjugated cel-mir-67. Dy547 signals were analyzed by flow cytometry at 24 h post-transfection. **(B)** Correlation summary of Dy547 mean fluorescence intensity (MFI) and transfection concentrations of cel-mir-67. **(C)** Live cell percentage was determined by trypan blue staining of cells transfected with indicated concentrations of Dy547-conjugated cel-mir-67 at indicated time points. **(D)** Growth curves of HeLa cells transfected with indicated concentrations of cel-mir-67. **(E–G)** HeLa cells transfected with 100 nM Dy547-conjugated cel-mir-67 and Dy547 signals were analyzed by flow cytometry at indicated time points after transfection **(E)**. Dy547 MFI was plotted against time to calculate its half-life **(F)**. Dy547 MFI in **(F)** was normalized to cell division **(G)**. Mock, HeLa cells transfected with 100 nM un-conjugated cel-mir-67.

We first examined the efficiency and dynamic range of transient transfection of miRNA mimics, following commonly used experimental conditions (Lim et al., 2005; Grimson et al., 2007; Baek et al., 2008; Selbach et al., 2008; Guo et al., 2010). We transfected HeLa cells with a *C. elegans* miRNA (cel-mir-67) conjugated with the Dy547 fluorophore at graded concentrations. The mature cel-mir-67 sequence bears no similarity with any mouse or human miRNAs and, therefore, is suitable for quantification. For the purpose of titrating, we tested a broad range of miRNA mimic concentrations (0.1–300 nM), including two concentrations that have been frequently used in various miRNA mechanism studies: 25 nM (Grimson et al., 2007; Baek et al., 2008) and 100 nM (Lim et al., 2005; Selbach et al., 2008; Guo et al., 2010). As shown in Figure 1A, transient transfection of miRNA mimic is very efficient, with measurable Dy547 signals at 0.5 nM. At higher concentrations the whole cell population was shifted toward a positive Dy547 signal, suggesting high transfection efficiency (Figure 1A). We observed an excellent dose-dependent response of Dy547 signal to transfection concentration of miRNA mimic without saturation at the highest concentration tested (300 nM), suggesting that the amount of miRNA mimic introduced into cultured cells can be controlled simply by adjusting the transfection concentration (Figure 1B). However, when transfected with a miRNA mimic at 150 nM or higher concentration, a significant fraction of cells started to die at 24 h post-transfection and viable cultures could not be maintained after that (Figure 1C). This suggests a limit to the amount of miRNA mimic that can be introduced into cultured cells. Moreover, cell growth was significantly retarded upon miRNA mimic transfection at 5 nM or higher concentrations (Figure 1D). Therefore, we decided to focus on 100 nM, a concentration frequently used in the literature (Lim et al., 2005; Selbach et al., 2008; Guo et al., 2010), in the following study.

Next we investigated the turnover time of transfected miRNA mimics by following the Dy547 signal in transfected cells for up to 72 h. At the 100 nM transfection concentration, the cellular miRNA mimic level peaked around 12 h, followed by a steady decrease over time, with a half-life of 28 h (Figures 1E–G). As the average doubling time of HeLa cells is 23 h (Jacobson and Ryan, 1982), the decrease in cellular miRNA mimic concentration is likely caused by cell division instead of active miRNA decay pathways (Figure 1F). Indeed, after normalizing the cellular levels of transfected cel-mir-67 to the doubling time of HeLa cells to remove the dilution effect caused by cell division, we found no active decay of the transfected cel-mir-67 (Figure 1G).

### Transfection of miRNA mimics at high concentrations leads to supraphysiological miRNA levels and accumulation of high molecular weight RNA species

**Figure 2:**
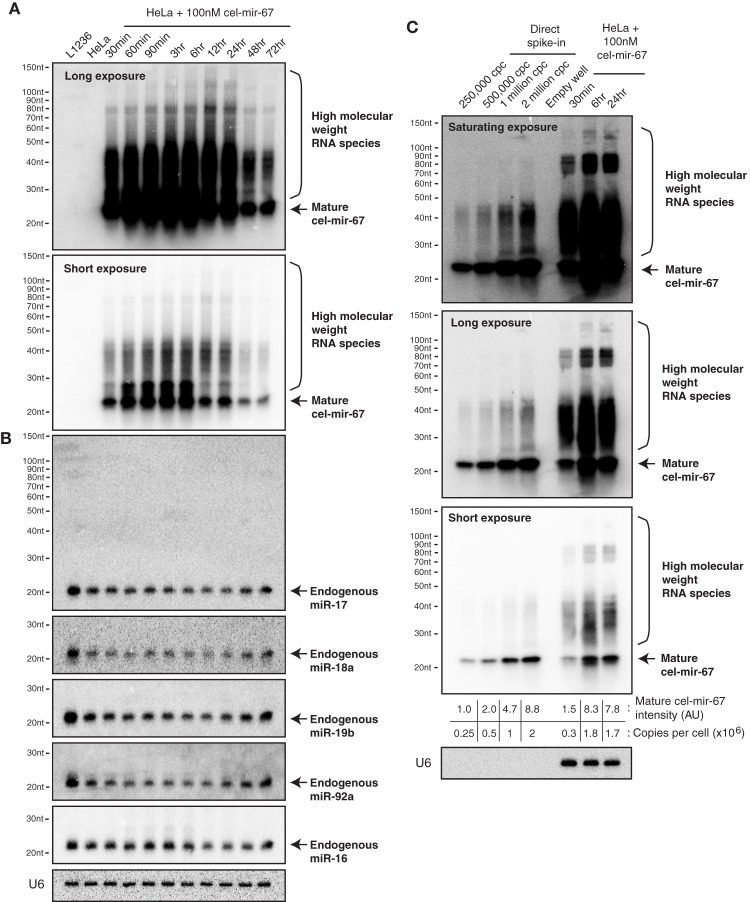
**Northern blot analysis of cel-mir-67 and endogenous miRNAs in HeLa cells transiently transfected with 100 nM cel-mir-67**. HeLa cells were transfected with 100 nM unconjugated cel-mir-67 and analyzed by Northern blot to detect cel-mir-67 **(A)** and endogenous miR-17, miR-18a, miR-19b, miR-92a, and miR-16 **(B)**. Arrows indicate mature cel-mir-67 and brackets indicate high molecular weight RNA species. L1236, a human Hodgkin's lymphoma cell line that expresses 2–3-fold more miR-17~92 than HeLa cells, was used as a universal reference throughout the manuscript. **(C)** The copy numbers of cel-mir-67 in transfected HeLa cells were estimated by comparing to graded amounts of the same miRNA mimic loaded directly into the Northern blot gel. Three different exposures of the same blot showed that the high molecular RNA species were generated by both the manufacturing process (before transfection) and cellular processes (after transfection), cpc, copies per cell. AU, arbitrary unit. The amount of transfected mir-cel-67 was estimated by comparing the mature signals of transfected mir-cel-67 to the spiked-in cel-mir-67.

To follow the behavior of transiently transfected miRNA mimic in cells in further details, we performed Northern blot analysis of HeLa cells transfected with 100 nM cel-mir-67 (without Dy547 conjugation) at various time points after transfection. As shown in Figure 2A, cel-mir-67 accumulated to significant levels as early as 30 min post-transfection, peaked at 3–6 h, and decreased drastically after 24 h. Strikingly, we observed the accumulation of RNA species larger than mature cel-mir-67, whose concentrations mirror that of mature cel-mir-67. These RNA species must be byproducts of transfected cel-miR-67, as they hybridized with the Northern blot probe, a cel-miR-67 antisense DNA oligonucleotide, and were absent in non-transfected HeLa cells and L1236 cells, a human Hodgkin's lymphoma cell line. Importantly, transient transfection of cel-mir-67 did not alter the expression level and size of endogenous miRNAs, including miR-17, miR-18a, miR-19b, miR-92a, and miR-16 (Figure 2B). Next, we estimated the copy numbers of cel-mir-67 mimic in transfected HeLa cells by comparing to graded amounts of the same miRNA mimic loaded directly into the Northern blot gel. Strikingly, transient transfection delivered 0.3 million copies of miRNA mimic per cell in 30 min post-transfection and this number reached 1.8 million at 6 and 24 h (Figure 2C), which is at least 10 times more than the estimated 100,000 copies per cell for all mature miRNAs in HeLa cells and mouse embryonic stem cells (Calabrese et al., 2007; Janas et al., 2012). Interestingly, the high molecular weight RNA species were already present in direct spike-in miRNA mimic samples, but appeared in higher amounts in miRNA mimic-transfected cells, suggesting that they were generated by both the manufacturing process and cellular processes (Figure 2C). The most abundant ones were in the size range of 25–50 nt (Figures 2A,C), which we decided to focus on in the following study.

**Figure 3:**
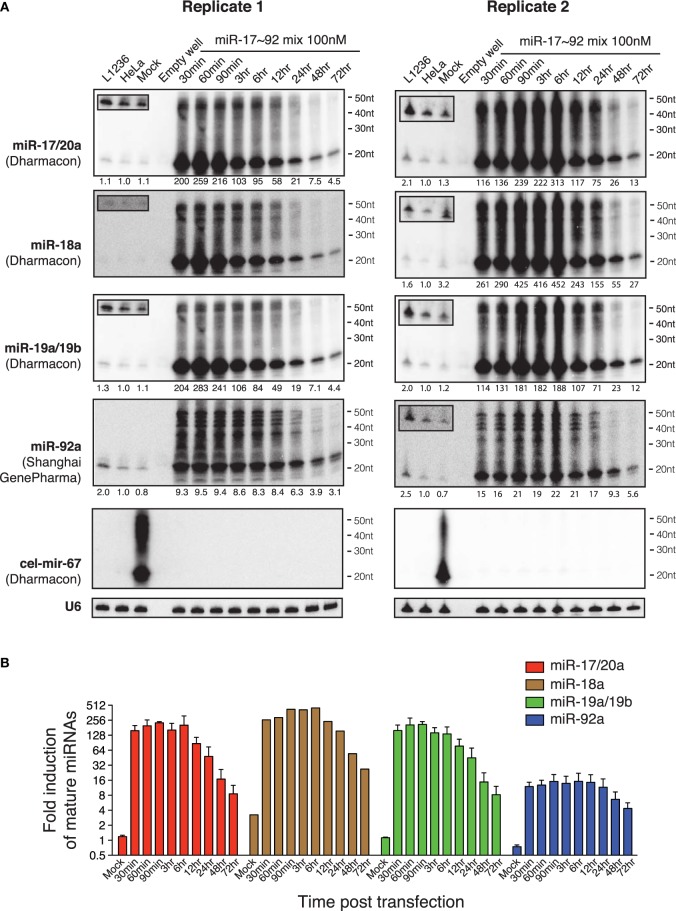
**Northern blot analysis of miR-17~92 miRNAs in HeLa cells transiently transfected with miR-17~92 mimics. (A)** HeLa cells were transfected with an equal molar mixture of miR-17, 18a, 19a, 20a, 19b, and 92a (16.7 nM each with a total transfection concentration of 100 nM) and analyzed by Northern blot to detect these miRNAs. Mixed probes were used for the detection of miRNAs in each subfamily. Numbers below each blot image indicate fold-induction of mature miRNAs (~22 nt). Mature miRNA expression levels in non-transfected HeLa cells were set as 1. L1236 cells and HeLa cells transfected with 100 nM cel-mir-67 (Mock) were used as controls. Small boxes in the upper left corner of Northern blots are longer exposures to show mature miRNAs in the same lanes. **(B)** Quantification of mature miRNA levels in **(A)**. Endogenous miR-18a in Replicate 1 was not detectable and was excluded from quantification.

Since cel-mir-67 is a *C. elegans* miRNA that has no homolog in mammalian species, we decided to perform the same experiments using microRNA-17~92 (miR-17~92), a miRNA cluster encoding six mature miRNAs (miR-17, miR-18a, miR-19a, miR-20a, miR-19b, and miR-92). miR-17~92 is broadly and abundantly expressed in mammalian tissues and cell lines (Lu et al., 2005; Kuchen et al., 2010). Mouse genetic studies from us and other investigators have demonstrated essential functions of these miRNAs in a large diversity of biological processes (He et al., 2005; Lu et al., 2007; Ventura et al., 2008; Xiao et al., 2008; Conkrite et al., 2011; De Pontual et al., 2011; Jiang et al., 2011; Baumjohann et al., 2013; Bian et al., 2013; Danielson et al., 2013; Jin et al., 2013; Kang et al., 2013; Khan et al., 2013). Importantly, a 3–5-fold increase in its expression in B cells was sufficient to drive lymphoma development (Jin et al., 2013, 2014). In experiments similar to those of cel-mir-67, we mixed the six miR-17~92 miRNAs at equal molar concentrations and transfected HeLa cells with a total concentration of 100 nM (16.7 nM for each miRNA). Interestingly, this led to accumulation of not only mature miR-17~92 miRNAs, but also high molecular weight RNA species similar to those observed for cel-mir-67 (Figures 2A, 3A), suggesting that the generation of high molecular weight RNAs is a general phenomenon associated with transient transfection of synthetic miRNA mimics. It is noteworthy that these miRNA mimics exhibited exactly the same behavior despite the fact that they were synthesized by two different companies (Dharmacon and Shanghai GenePharma) in two different countries.

Our previous study demonstrated that the miR-17~92 family miRNAs are expressed at similar levels in HeLa cells and in murine B cells (Xiao et al., 2008). When transfected into HeLa cells at individual concentrations of 16.7 nM, they achieved a 10-20-fold (miR-92) or 200–400-fold (miR-17/20a, 18a, 19a/19b) overexpression (Figure 3), far exceeding the 3–5-fold increase that was sufficient to drive B cell lymphoma development in mice (Jin et al., 2013), as well as the 2–36-fold increase found in biopsies of human Burkitt's lymphomas, which consistently exhibit activation of the c-Myc-miR-17~92 axis (Schmitz et al., 2012). Studies from us and other investigators estimated that miR-17~92 miRNAs are expressed at a few thousand copies per cell (Mukherji et al., 2011; Bosson et al., 2014). Therefore, transient transfection at individual concentrations of 16.7 nM brings the total amount of miR-17~92 miRNAs to a million copies per cell, which is similar to the estimated copy number of cel-mir-67 when transfected at 100 nM (Figure 2C). Taken together, these results demonstrate that commonly used transient transfection concentrations (25 and 100 nM) bring cellular miRNAs to supraphysiological levels and, thereby, may cause saturation of the miRNA system.

### Transfection of miRNA mimics at low concentrations is sufficient to achieve pathological miRNA levels

**Figure 4:**
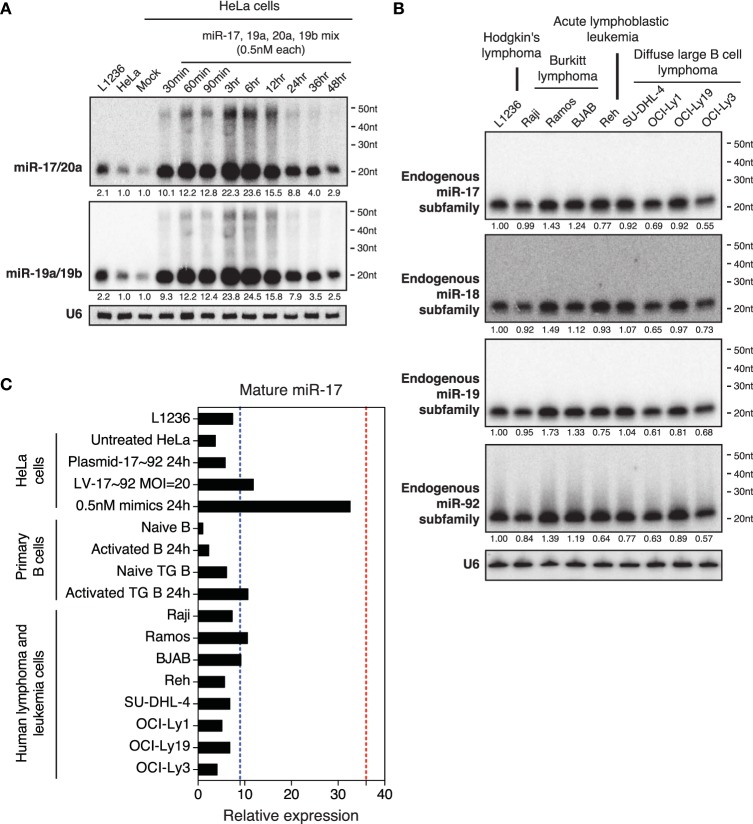
**Northern blot analysis of miRNAs in HeLa cells transiently transfected with low concentrations of miR-17~92. (A)** HeLa cells were transfected with an equal molar mixture of miR-17, 19a, 20a, 19b (0.5 nM each with a total transfection concentration of 2 nM) and analyzed by Northern blot to detect these miRNAs. Mixed probes were used for the detection of miRNAs in each family. Numbers below blots indicate fold increase of mature miRNA levels. Mature miRNA expression levels in non-transfected HeLa cells were set as 1. **(B)** Northern blot analysis of endogenous miR-17~92 levels in nine human lymphoma or leukemia cell lines. Mixed probes were used for the detection of miRNAs in each family. Numbers below blots indicate fold increase of mature miRNA levels. Mature miRNA expression levels in L1236 were set as 1. **(C)** Summary of miR-17 expression levels in samples used in this study. Results for primary B cells, lentivirally transduced (LV-17~92) and plasmid transfected (Plasmid-17~92) HeLa cells were from Figure 5. Red and blue dashed lines indicate the maximal and mean miR-17 expression levels in human Burkitt's lymphoma patients, respectively (Schmitz et al., 2012). Mature miR-17 expression level in naïve B cells was set as 1.

To imitate miR-17~92 expression levels found in the biopsies of human Burkitt's lymphomas (2–36-fold) (Schmitz et al., 2012), we transfected an equal molar mix of miR-17, 19a, 20a, and 19b at 0.5nM for each miRNA. miR-17 and miR-20a belong to the miR-17 family, while miR-19a and miR-19b belong to the miR-19 family. As members in these families share identical seed regions and differ from each other by only 1–3 nucleotides, they cannot be easily distinguished by Northern blot. Therefore, we mixed probes for members of each family together and performed Northern blot with the mixed probes. The transfection concentration for each family was 1 nM, which is 25–100-fold lower than what was commonly used in the literature (Lim et al., 2005; Grimson et al., 2007; Baek et al., 2008; Selbach et al., 2008; Guo et al., 2010). At this transfection concentration, the cellular levels of miR-17 and miR-19 family miRNAs reached 10-fold of endogenous levels in 30 min post-transfection, peaked around 23-fold at 3–6 h, and started to decline afterwards (Figure 4A). Similar to transient transfection at higher concentrations (Figures 2, 3), we still observed high molecular weight RNA species in transfected cells, though they represented a much lower percentage of Northern blot signals (Figure 2C). To directly compare the degree of overexpression achieved by this low transfection concentration with miR-17~92 overexpression in lymphomas and leukemias, we measured the endogenous miR-17~92 levels in nine human lymphoma and leukemia cell lines. The maximal miR-17~92 expression level in these cell lines was about 10-fold of that found in naïve B cells (Figures 4B,C), consistent with the 2–36-fold overexpression found in biopsies from human Burkitt's lymphoma patients (Schmitz et al., 2012) (Figure 4C). Importantly, none of these cell lines express the high molecular weight RNA species found in transiently transfected HeLa cells (Figure 4B). Collectively, these results demonstrate that transient transfection with 1 nM miRNA mimics is sufficient to achieve expression levels comparable to those found in primary human lymphomas and leukemia, and minimizes the generation of high molecular weight RNA species.

### Lentiviral transduction, plasmid transfection and transgene achieve physiological miRNA overexpression and efficiently suppress target gene expression

**Figure 5:**
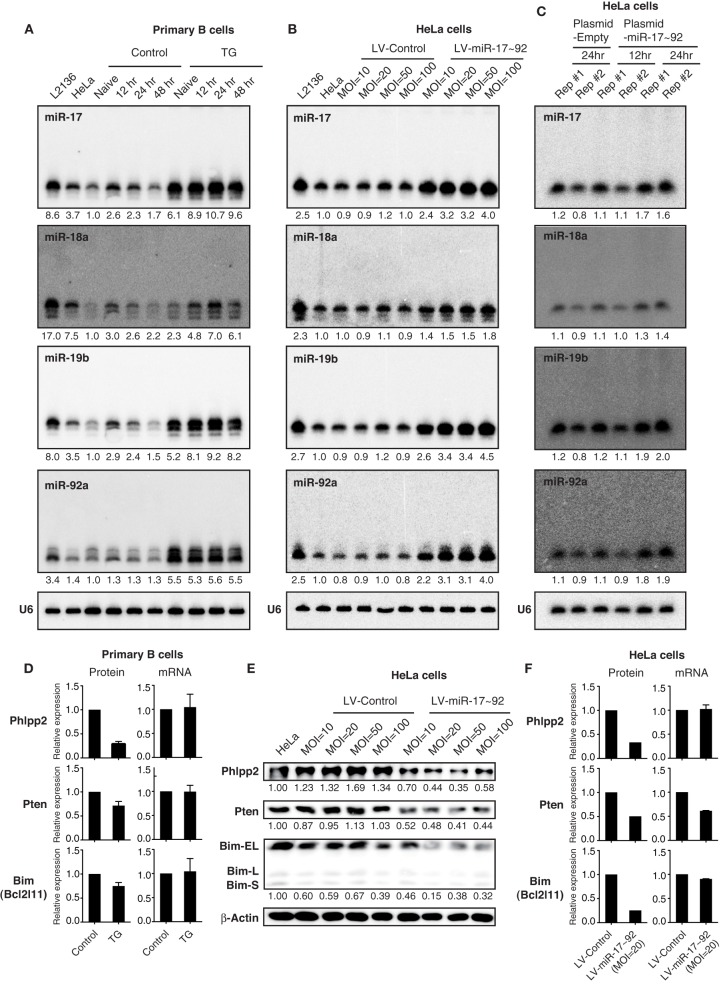
**The effect of transgenic and lentiviral miR-17~92 expression on target genes. (A–C)** Northern blot analysis of miR-17~92 expression. Primary B cells were purified from CD19-Cre (Control) and miR-17~92 Tg/Tg; CD19-Cre (TG) mice, stimulated *in vitro* for indicated amounts of time **(A)**. HeLa cells were stably transduced with control or miR-17~92 expressing lentiviruses at indicated MOI (Multiplicity of infection) **(B)**. HeLa cells were transfected with empty plasmid or miR-17~92-expressing plasmid (Plasmid-miR-17~92) and harvested at indicated time points **(C)**. Numbers below blots indicate miR-17~92 expression normalized to U6. miRNA/U6 ratios in naïve control B cells **(A)**, non-transduced HeLa cells **(B)**, and empty plasmid transfected HeLa **(C)** were arbitrarily set as 1. **(D)** Quantification of protein and mRNA levels of target genes in primary B cells (*n* = 4). The representative western blot results were presented previously (Jin et al., 2013). **(E)** Western blot analysis of miR-17~92 target gene expression in lentivirally transduced HeLa cells. Different MOI are indicated. Numbers below blots indicate target gene expression normalized to β-Actin. The ratios in parental HeLa cells were arbitrarily set as 1. **(F)** Quantification of protein and mRNA levels of target genes in lentivirally transduced HeLa cells. **(D–F)** Target gene protein and mRNA levels were determined by Western blot and qRT-PCR, respectively.

Other commonly used methods to ectopically express miRNAs are through lentiviral infection, retroviral infection, plasmid transfection and transgene expression. We next examined miRNA expression by these methods, focusing on expression levels and the appearance of high molecular weight RNA species. We have previously generated a conditional miR-17~92 transgene (termed miR-17~92 Tg), whose expression can be turned on in B cells by CD19-Cre, and showed that the resulting transgenic mice (TG) developed lymphomas with high penetrance (Jin et al., 2013). We examined miR-17~92 expression in primary B cells isolated from these mice in the presence and absence of stimulation. As shown in Figure 5A, transgenic miR-17~92 expression led to a 2–6-fold increase in the amounts of these miRNAs. On the other hand, stable transduction of HeLa cells with a miR-17~92 expressing lentivirus at MOI (multiplicity of infection) of 10–100 led to a 2–5-fold, while transient transfection of the same cells with a miR-17~92 expressing plasmid led to a 2-fold increase in the expression of these miRNAs. Therefore, these three methods achieved similar fold increases in miR-17~92 expression (Figures 5A–C), which were in the same range as that observed in human lymphoma and leukemia cell lines (Figure 4C). Importantly, in none of these experimental conditions did we see the high molecular weight RNA species that are characteristic of transient transfection of miRNA mimics (Figures 5A–C). Lentiviruses, plasmids, and transgenes used in this study were designed to include pri-miRNAs and their flanking regions (Xiao et al., 2008), which are critical for their biogenesis and expression (Guil and Cáceres, 2007; Du et al., 2015). In addition, transgenes and lentiviruses are integrated into the host cell genome, and mature miRNA production by these methods likely follows the same biogenesis pathway as endogenous miRNAs. This may explain the limited fold increase achieved by these methods, as well as the absence of high molecular weight RNA species.

Next we examined the effect of lentivirally expressed miR-17~92 on its target gene expression by Western blot. Similar to transgenic miR-17~92 expression in B cells (Jin et al., 2013), a 2–5-fold increase in miR-17~92 expression in HeLa cells was sufficient to suppress the protein output of previously validated miR-17~92 target genes (Phlpp2, Pten, and Bim) (Figure 5E) (Ventura et al., 2008; Xiao et al., 2008; Rao et al., 2012; Jin et al., 2013; Kang et al., 2013; Xu et al., 2015). The percentage reduction in target gene protein levels was similar to, or even higher than, that achieved by transgenic miR-17~92 expression (Figures 5D–F), which is able to drive lymphoma development with high incidence (Jin et al., 2013). We also examined the effect of transgenic and lentiviral miR-17~92 expression on target gene mRNA levels. While transgenic miR-17~92 expression predominantly suppressed target gene expression at the translation level (Figure 5D), the effect of lentiviral miR-17~92 expression was target gene-specific: it suppressed the expression of Phlpp2 and Bim by translation repression, and Pten mainly through mRNA degradation (Figure 5F). Taken together, in spite of some minor differences, miRNA expression by lentivirus and transgene achieves similar expression levels and exerts comparable effects on target genes.

### Transfection of miRNA mimics at high concentrations causes non–specific changes in gene expression

**Figure 6:**
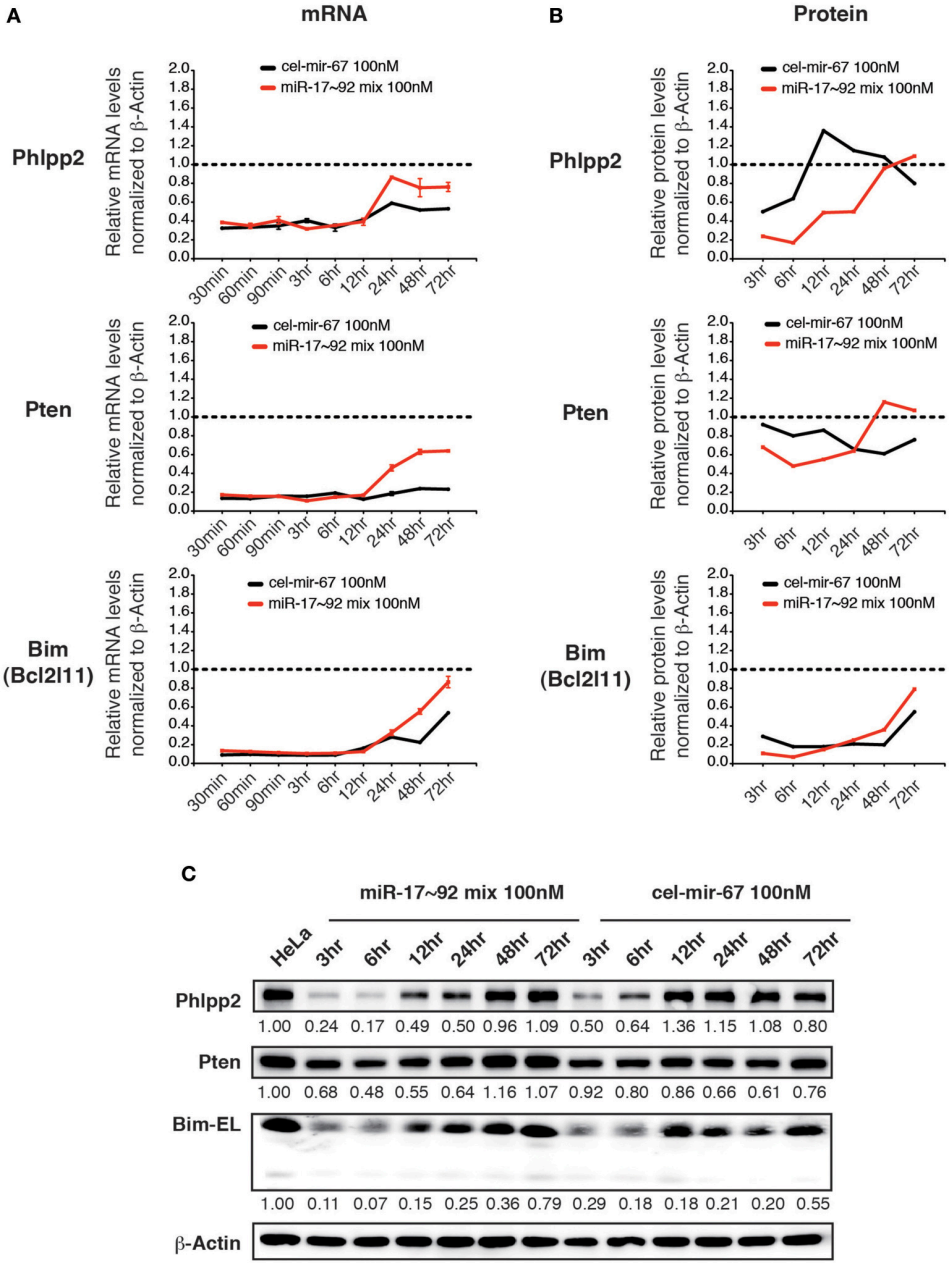
**The effect of high concentration transient transfection on target gene expression**. HeLa cells were transiently transfected with miRNA mimics. Target gene mRNA and protein levels were determined by qRT-PCR and Western blot at indicated time points and normalized to β-Actin. The target gene/β-Actin ratios of non-transfected HeLa cells were arbitrarily set as 1 (dashed line). miR-17~92 mix 100 nM contains an equal molar mixture of miR-17~92 miRNAs at 16.7 nM for each miRNA with a total concentration of 100 nM. **(A,B)** Summary of mRNA and protein quantification results. **(C)** Representative Western blots.

We then examined the effect of transiently transfected miR-17~92 mimics on the same group of target genes. Upon transfection with 100 nM miR-17~92 mimics, the mRNA levels of all three target genes were reduced by 60~80% at 30 min post-transfection, and started to recover after 12 h (Figure 6A). Surprisingly, transient transfection of cel-mir-67 mimic caused similar degree of mRNA reduction for the same set of genes, and their recovery was even slower (Figure 6A). In addition, transient transfection of both miR-17~92 and cel-mir-67 mimics led to reduction in protein levels of these target genes, though the kinetics differs for different target genes (Figures 6B,C). This is unexpected because cel-mir-67 bears no sequence similarity to any mammalian miRNAs including miR-17~92. We searched these target mRNAs for potential cel-mir-67 binding sites and found only one 7-mer site in the Pten 3′UTR and none in the Bim and Phlpp2 mRNAs. We cannot fully exclude the possibility that the reduction in the Pten mRNA and protein levels was mediated by the 7-mer cel-mir-67 binding site in its 3′UTR, but the decrease in Bim and Phlpp2 expression was likely due to non-specific effects. Therefore, transient transfection of miRNA mimics at high concentrations (100 nM) can alter gene expression in a non-specific manner.

### Transfection of miRNA mimics does not induce interferon responses

**Figure 7:**
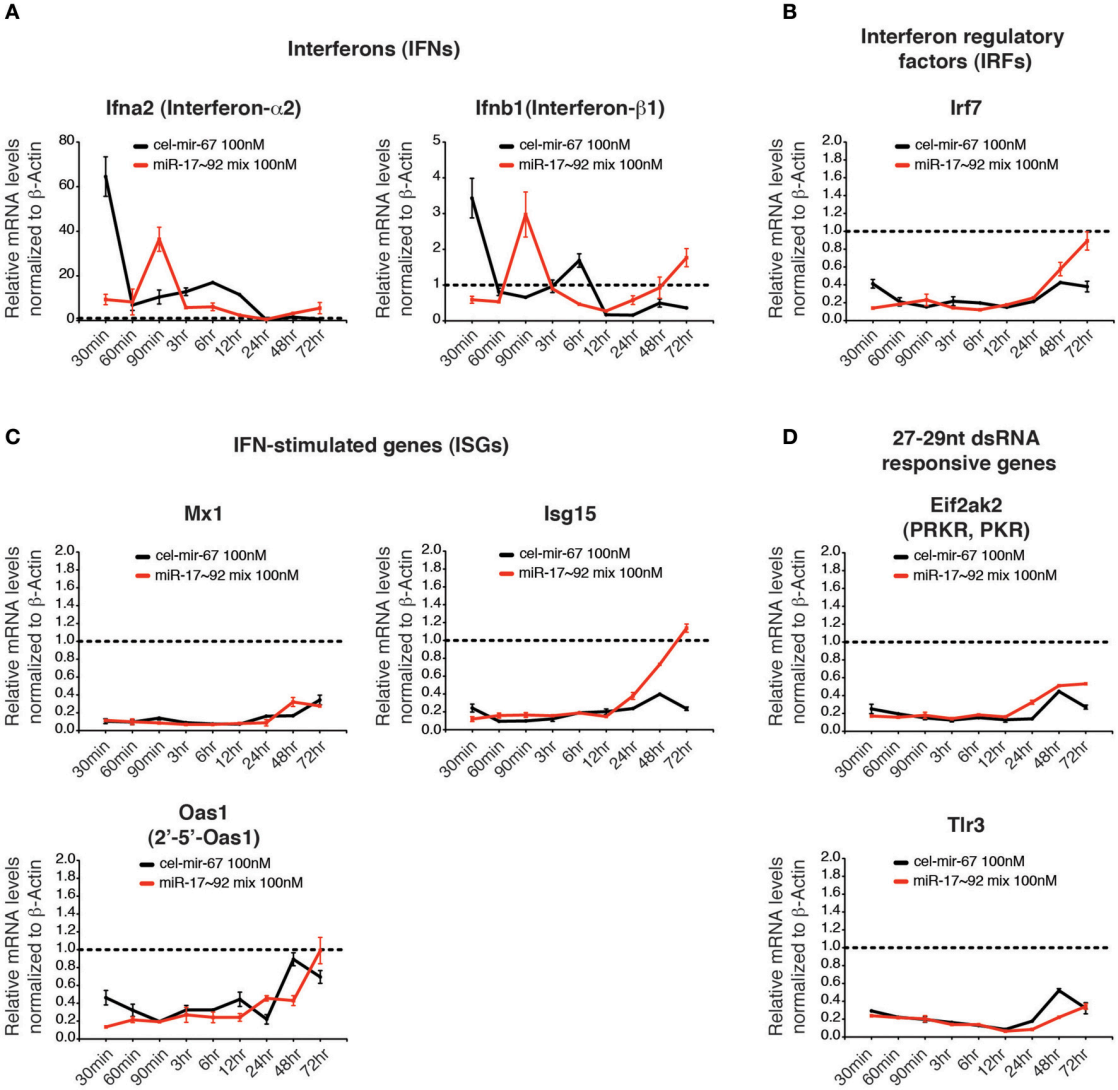
**The effect of high concentration transient transfection on innate immune responses**. The mRNA levels of genes involved in innate immune response to dsRNA were determined by qRT-PCR and normalized to β-Actin. The target gene/β-Actin ratios of non-transfected HeLa cells were arbitrarily set as 1 (dashed line). Type I interferons **(A)**, interferon regulatory factors **(B)**, interferon stimulated genes **(C)** and genes specifically responding to 27–29 nt long dsRNA **(D)** were investigated.

Previous studies showed that double-stranded RNAs (dsRNAs) induce innate immune responses, especially the interferon responses (Wang and Carmichael, 2004), which may lead to the non-specific gene suppression caused by transient transfection of miRNA mimics. We investigated several innate immune responsive genes whose expressions are highly induced by long dsRNAs, including interferons (IFNs: Ifna2 and Ifnb1), an interferon regulatory factor (IRF: Irf7), and interferon-stimulated genes (ISGs: Mx1, Oas1, Isg15) (Figures 7A–C) (Dewitte-Orr et al., 2009). We also examined Tlr3 and Eif2ak2, whose expression is specifically induced by transient transfection of 27–29 bp long dsRNAs (Reynolds et al., 2006) (Figure 7D). Among all genes examined, only interferon-α2 (Ifna2) and interferon-β1 (Ifnb1) were induced at early time points of transfection, but their induction was quickly resolved (Figure 7A). Importantly, the transient induction of IFNs did not trigger the expression of other innate immune response genes. On the contrary, most of the dsRNA-responsive genes examined were suppressed by transient transfection of miRNA mimics (Figures 7B–D). Therefore, it is unlikely that non-specific suppression of target gene expression caused by transient transfection of high concentration miRNA mimics is due to induction of innate immune responses.

### Transfection of miRNA mimics at low concentrations fails to suppress target gene expression

**Figure 8:**
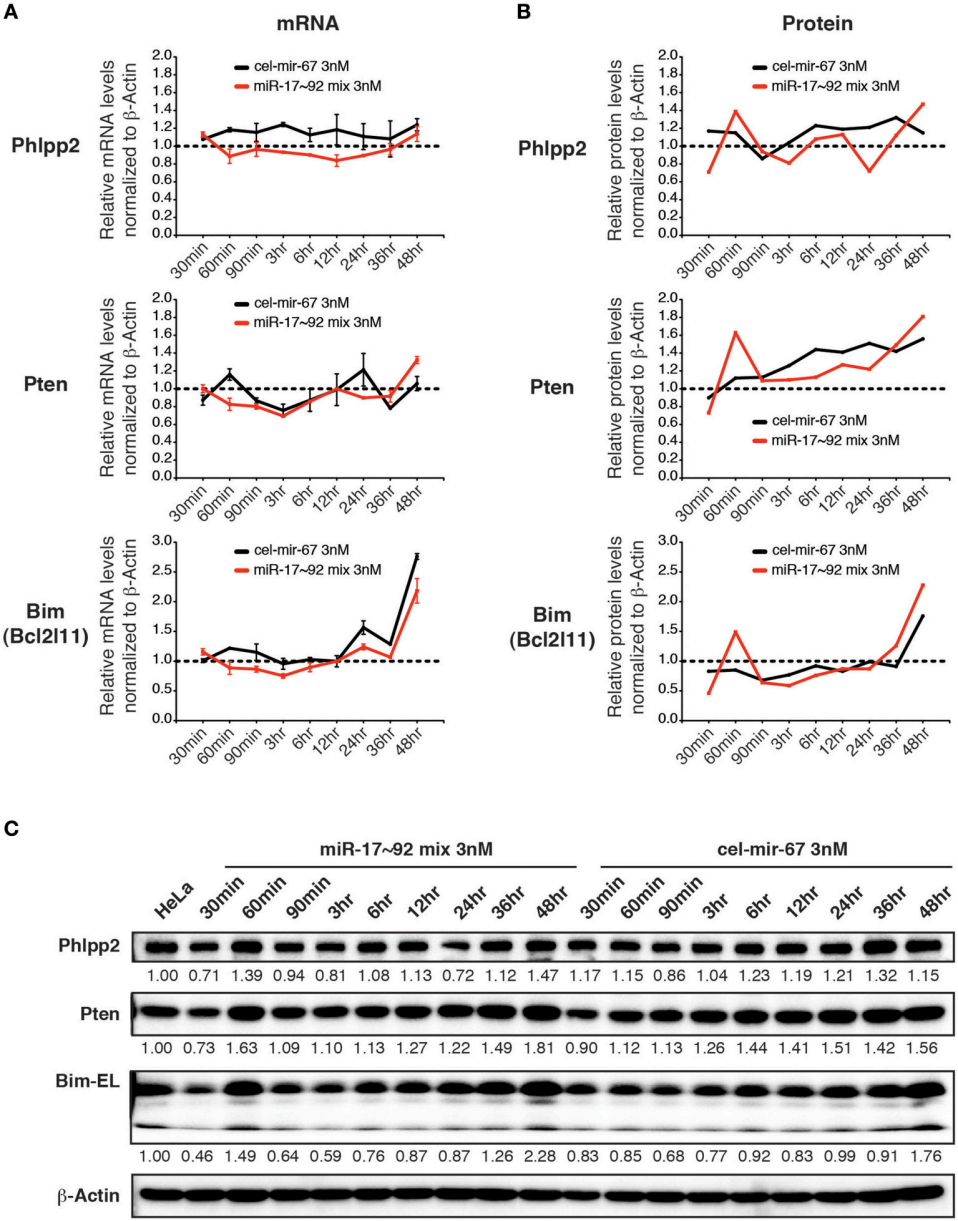
**The effect of low concentration transient transfection on target gene expression**. HeLa cells were transfected with an equal molar mixture of miR-17~92 miRNAs at 0.5 nM for each miRNA with a total concentration of 3 nM. Target gene mRNA and protein levels were determined by qRT-PCR and Western blot at indicated time points and normalized to β-Actin. The target gene/β-Actin ratios of non-transfected HeLa cells were arbitrarily set as 1 (dashed line). **(A,B)** Summary of mRNA and protein quantification results. **(C)** Representative Western blots.

We next examined the effect of transient transfection of low concentrations of miRNA mimics on target genes. For this purpose, we transfected HeLa cells with a mixture of miR-17~92 miRNA mimics at 0.5 nM for each member (3 nM in total) and measured target gene mRNA and protein levels. Importantly, this low concentration transfection achieved a 4–20-fold increase in miR-17~92 expression (Figure 4A). This amount of overexpression was able to suppress target gene expression when introduced by transgene, lentivirus, or plasmid transfection (Figure 5D) (Xiao et al., 2008; Jin et al., 2013; Kang et al., 2013). In stark contrast, the 4–20-fold increase in miR-17~92 expression brought about by transient transfection of low concentrations of miRNA mimics was not sufficient to suppress target gene expression (Figures 8A–C). Therefore, transient transfection of miRNA mimics at high concentrations led to non-specific alterations in gene expression, while at low concentrations was not effective in suppressing target gene expression.

### Supraphysiological overexpression and high molecular weight RNA species is a general phenomenon associated with miRNA mimic transfection

**Figure 9:**
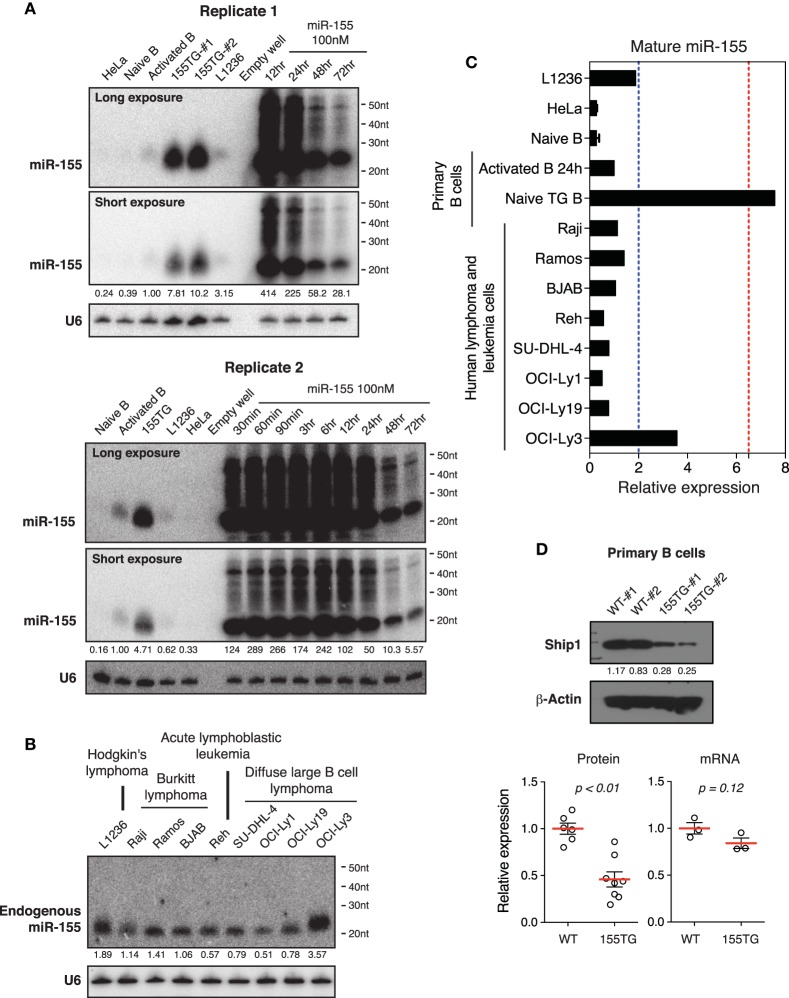
**The effect of transient transfection and transgenic expression of miR-155. (A)** Northern blot analysis of miR-155 expression in HeLa cells transiently transfected with a miR-155 mimic, and in primary B cells from wild type and miR-155 transgenic mice (155TG). Two different exposures of the same blot are included. **(B)** Northern blot analysis of endogenous miR-155 expression in nine human lymphoma or leukemia cell lines. **(C)** Summary of miR-155 Northern blots in **(A,B)**. Mature miR-155 expression in *in vitro* activated B cells was arbitrarily set as 1 in **(A–C)**. Red and blue dashed lines in **(C)** indicate the maximal and mean miR-155 expression levels in human lymphoma patients, respectively. This was estimated from absolute copy numbers of miR-155 in activated B cells (data not shown) compared to that in lymphoma patients (Eis et al., 2005). **(D)** Ship1 expression in miR-155 transgenic B cells. Upper panel, a representative Western blot. Lower panel, bar graphs summarizing Ship1 protein and mRNA levels in miR-155 transgenic B cells as determined by Western blot and Microarray. Each circle indicates an independent biologic sample.

To determine whether our observations are a general phenomenon of transient transfection of miRNA mimics, we investigated miR-155, a lymphocyte specific miRNA. miR-155 is expressed at relatively low levels in naïve B cells, and is significantly induced upon B cell activation (Rodriguez et al., 2007; Thai et al., 2007). Similar to miR-17~92, miR-155 is frequently up-regulated in human B cell lymphomas (Eis et al., 2005; Rai et al., 2008), and transgenic expression of miR-155 is sufficient to drive B cell lymphoma development in mice (Costinean et al., 2006). In addition, transient transfection of miR-155 mimic into HeLa cells was used as a model system to study miRNA mechanism of action in mammalian cells (Guo et al., 2010). Under the same experimental conditions, the peak cellular concentrations of miR-155 reached 200–400-fold of that found in activated B cells (Figure 9A). This level of overexpression was about 50-fold of that achieved by transgenic miR-155 expression in B cells, which was sufficient to drive lymphoma development (Costinean et al., 2006). Similar to miR-17~92 and cel-mir-67 mimic, transient transfection of miR-155 mimic also caused accumulation of high molecular weight RNA species (Figure 9A). We next examined the endogenous levels of miR-155 in our panel of nine human lymphoma and leukemia cell lines. As shown in Figures 9B,C, the highest miR-155 expression in these cell lines was a few fold of that found in activated B cells. Consistent with miR-17~92 results, there was no miR-155-specific high molecular weight RNA species in activated B cells, transgenic B cells, and lymphoma and leukemia cells (Figures 9A,B). Lastly, we examined the effect of transgenic miR-155 expression on its functional target gene, Ship1 (O'connell et al., 2009; Pedersen et al., 2009). While a previous study showed that the Ship1 protein was upregulated in activated B cells deficient of miR-155 (O'connell et al., 2009), it was significantly downregulated in miR-155 transgenic (termed 155TG) B cells, accompanied by a marginal reduction in its mRNA level (Figure 9D). These results suggest that the predominant effect of miR-155 on Ship1 is exerted at the translation level (Figure 9D). Taken together, transient transfection of a miR-155 mimic at 100 nM led to a few hundred fold increase in the mature miRNA level and accumulation of high molecular weight RNA species, which are similar to miR-17~92 and cel-mir-67 mimics.

### Mutated guide strands and unnatural passenger strands accumulate in miRNA mimic transfected cells

**Figure 10:**
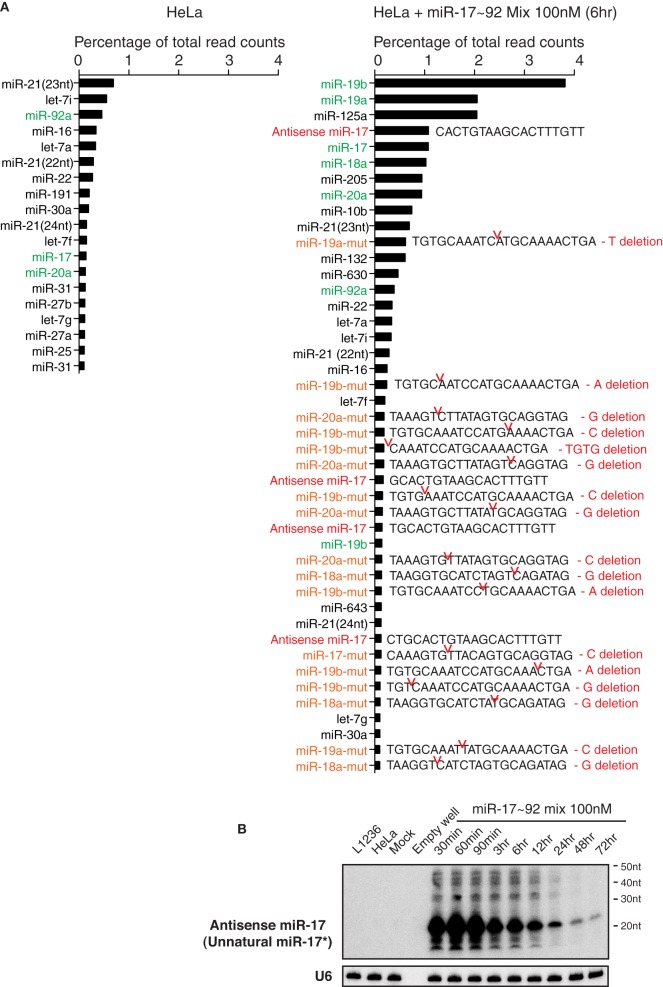
**Transient transfection of miRNA mimics led to the accumulation of mutated miRNAs and unnatural miRNA passenger strands. (A)** Non-transfected HeLa cells and HeLa cells transfected with miR-17~92 miRNA mimics were analyzed by small RNA deep sequencing at 6 h after transfection (the same sample as in Figure 3A, replicate 1, miR-17~92 mix 100 nM). Mature miRNAs, antisense miRNAs, and their mutant forms with abundance higher than 0.1% of total reads are listed. Note that non-transfected HeLa cells do not express antisense miRNAs or mutant miRNAs at levels higher than this cut off, while HeLa cells transfected with miR-17~92 mix contain many of these species. Antisense miR-17 sequences (marked in red) are perfectly complementary to miR-17, and differ from miR-17^*^, the endogenous form of miR-17 passenger strand, by a few nucleotides. miR-17~92 miRNAs are marked in green, and their mutant forms are marked in orange. Sequences of the latter are included. Red notches indicate the location of deletion, with deleted nucleotides presented at the right. **(B)** Northern blot analysis of antisense miR-17 (as indicated in the right panel of **A**) in transfected HeLa cells. The blot from Figure 3A Replicate 1 was hybridized with a probe specific for antisense miR-17.

We next performed small RNA deep sequencing analysis of non-transfected HeLa cells and HeLa cells transiently transfected with miR-17~92 miRNA mimics to investigate the identity of high molecular weight RNA species. Our library preparation strategy allowed us to capture both mature miRNAs and the high molecular weight RNA species. The total read counts of non-transfected HeLa cells (4.14 × 10^6^) and miR-17~92 miRNA mimics-transfected HeLa cells (4.08 × 10^6^) were almost identical, so that we can directly compare the read counts of individual RNA species in these two samples. Consistent with the Northern blot results (Figure 3), there was a significant increase in the read counts for miR-17~92 miRNAs in miRNA mimics-transfected HeLa cells (Figure 10A). Surprisingly, mutated forms of miR-17~92 miRNAs were present in high abundance in miRNA mimics-transfected HeLa cells, while completely absent in non-transfected HeLa cells. Most of the mutations were single nucleotide deletion, but trimmed forms were also found. In addition, sequences perfectly antisense to mature miR-17 (termed antisense miR-17) were found in high abundance, probably arising from the passenger strand of the miR-17 mimic. These antisense miR-17 sequences differ from miR-17^*^, the endogenous miR-17 passenger strand, by a few nucleotides (Figure 10A). The presence of antisense miR-17 (unnatural miR-17^*^) in transfected HeLa cells was confirmed by Northern blot analysis (Figure 10B). We speculate that the mutated miR-17~92 miRNAs, together with antisense miR-17, may cause the non-specific alterations in gene expression in miRNA mimics-transfected HeLa cells (Figure 6). In line with this, a recent report showed that passenger strands derived from miRNA mimics accumulated in transiently transfected cells and were potent in suppressing their target genes (Søkilde et al., 2015).

### Characterization of high molecular weight RNA species in miRNA mimic transfected cells

**Figure 11:**
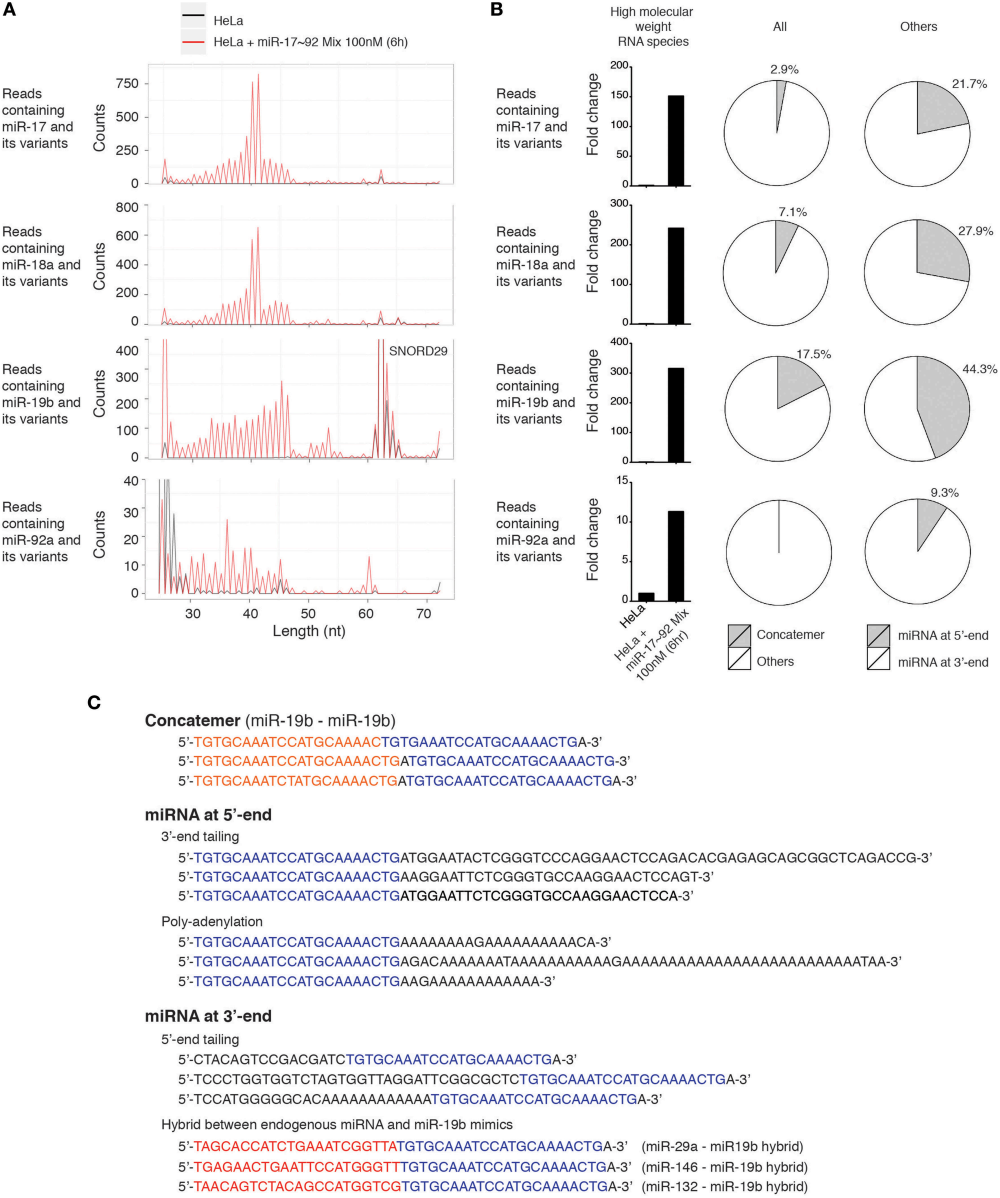
**Characterization of high molecular weight RNA species**. **(A)** Length distribution of high molecular weight RNA species identified in the small RNA deep sequencing data of Figure 10. Each peak represents the read count of high molecular weight RNA species of indicated length. SNORD29 contains sequence region highly homologous to miR-19b and is detected in both non-transfected and miR-17~92 mix-transfected HeLa cells. **(B)** Fold change of high molecular weight RNA species in miR-17~92 mix-transfected HeLa cells. The abundance in non-transfected HeLa cells was arbitrarily set as 1. RNA species of 30–60 nt are included in the calculation to exclude SNORD29. The relative abundance of concatemer, 5′-tailing, and 3′-tailing are presented in pie charts. **(C)** Representatives of high molecular weight RNA species, using miR-19-containing sequences as examples.

Since miR-17~92 miRNA mimics were frequently mutated and trimmed in transfected cells, and probes used in our Northern blot experiments can potentially hybridize with RNA species with imperfect complementarity, we searched the deep sequencing data for high molecular weight RNA species containing miR-17~92 miRNA sequences, allowing a few nucleotide mismatches. Consistent with Northern blot results, miR-17~92 miRNA mimics-transfected HeLa cells did contain high molecular weight RNA species harboring individual miR-17~92 miRNA sequences. The length distribution of these long reads is consistent with that revealed by Northern blot (Figures 3A, 11A), and they are absent in non-transfected HeLa cells (Figure 11B). Most of these long reads contain non-templated addition of nucleotides (tailing) to the 5′ or 3′ end of miR-17~92 miRNAs. In addition, miRNA concatemers, hybrids between miR-17~92 miRNAs and other endogenous miRNAs, and polyadenylated miR-17~92 miRNAs were also identified, but their abundance was much lower than those with 5′- and 3′-end tailing (Figures 11B,C). Therefore, our small RNA deep sequencing analysis confirmed the accumulation of high molecular weight RNA species in miRNA mimics-transfected cells, which were a heterogeneous mixture of several classes of RNA species that may be generated by concatemerization, 5′- and 3′-end tailing of miRNA mimics.

## Discussion

The present study demonstrates that transient transfection of miRNA mimics into HeLa cells using a commonly used protocol and transfection concentration led to accumulation of high molecular weight RNA species, a few hundred fold increase in mature miRNA levels, and an estimated cellular concentration of 1 million copies per cell, which is 10 times of the whole mature miRNA pool in a cell. In contrast, transgenic expression, plasmid transfection and lentiviral transduction of the same miRNAs achieved less than 10-fold overexpression, which was sufficient to suppress target gene expression and drive lymphoma development. In addition, the levels of endogenous overexpression of these miRNAs in a panel of human lymphoma and leukemia cell lines were similar to these achieved by transgene-, plasmid-, and lentivirus-driven expression, with no accumulation of high molecular weight RNA species in any of these four experimental conditions. Furthermore, transient transfection of miRNA mimics at high concentrations altered gene expression in a non-specific manner, while at low concentrations failed to efficiently suppress target gene expression. The non-specific effects of miRNA mimics may be caused by the supraphysiological levels of mature miRNAs and the accumulation of mutated guide strands, unnatural forms of passenger strands, and high molecular weight RNA species generated by concatemerization, 5′- and 3′-end tailing of miRNA mimics. These findings call into question the physiological relevance of the large volume of studies employing the transient transfection approach to investigate the functions and mechanisms of individual miRNAs.

A case in point comes from recent studies employing the transient transfection approach which concluded that mammalian miRNAs predominantly act to decrease target mRNA levels rather than decreasing translation efficiency (Guo et al., 2010). Re-analysis of this data set revealed that translation repression precedes mRNA degradation upon miRNA targeting (Larsson and Nadon, 2013). The latter finding was consistent with previous studies of miR-430 during the early stage of embryogenesis in zebrafish. miRNAs are essentially absent from fertilized zebrafish eggs (Chen et al., 2005). Around 4 h post-fertilization (4 hpf), a zebrafish-specific miR-430 family is expressed (Chen et al., 2005; Giraldez et al., 2006). This miRNA family is very unusual in that it has ~100 copies of genes distributed over three clusters in the zebrafish genome (Thatcher et al., 2008). The mature miR-430 miRNAs reach millions of copies in a few hours after fertilization and remain as the only miRNAs expressed until 12 hpf, when a few other miRNAs begin to be expressed (Chen et al., 2005). Ribosome profiling studies showed that miR-430 miRNAs promote translation repression followed by deadenylation and degradation of maternal mRNAs (Bazzini et al., 2012). The temporal and quantitative nature of miR-430 induction is unique for the early embryogenesis of zebrafish. Extensive deep sequencing analysis of mammalian tissues and cells has, to date, not found any miRNAs with similar abundance and dominance in any cellular contexts examined (Lu et al., 2005; Kuchen et al., 2010). It has been estimated that mammalian cells often express 100–200 different species of miRNAs (Kuchen et al., 2010), with a total amount of 1–2 × 10^5^ copies of mature miRNAs in a cell (Calabrese et al., 2007; Janas et al., 2012). The most abundant miRNAs are expressed at the level of ~2 × 10^4^ copies per cell (Neilson et al., 2007; Kuchen et al., 2010). Therefore, it is very unlikely that the million-copy-per-cell expression level of miR-430 in zebrafish embryos, as well as those achieved by transient transfection of high concentrations of miRNA mimics into mammalian cells, represents any mammalian miRNAs under physiological or pathological conditions. The translation repression followed by mRNA degradation model of miRNA mechanism of action, which was originally proposed based on the study of zebrafish miR-430, may not be universally applicable to mammalian miRNAs under physiological conditions. In line with this, an extensive survey of functionally relevant target genes validated in 77 miRNA mutant mice showed that the predominant mode of target gene suppression by miRNA is cell type-, miRNA- and target gene-dependent, with varying contributions from translation repression and mRNA degradation, suggesting that different miRNA-target gene interactions are governed by different mechanisms (Jin and Xiao, 2015).

The high molecular weight RNA species present in cells transfected with high concentrations of miRNA mimics are of great interest. Our small RNA deep sequencing analysis showed that they are a heterogeneous mixture of several classes of RNA species that are generated by concatemerization, 5′- and 3′ -end tailing of miRNA mimics. Northern blot analysis revealed that low concentrations of these high molecular weight RNA species were already present in miRNA mimics before transfection, likely generated during the manufacturing process. Their amounts were drastically increased upon transfection into HeLa cells, suggesting the existence of cellular mechanisms that convert miRNA mimics into longer forms. Previous studies have shown frequent modifications of endogenous miRNAs, including RNA editing, RNA methylation, uridylation, and adenylation (Ameres and Zamore, 2013; Ha and Kim, 2014). In addition, extensive complementarity between a target mRNA and an Ago-bound miRNA triggers miRNA tailing and trimming, leading to the generation of high molecular weight RNA species in the same size range as these observed in this study (Ameres et al., 2010). The exact molecular pathways that convert miRNA mimics into high molecular weight RNA species and the functions of these pathways under physiological and pathological conditions warrant future investigation.

Based on our observations, several recommendations can be made for miRNA studies in the future. First, lentiviral, retroviral, and genetic approaches are preferred over transient transfection to alter cellular concentrations of individual miRNAs. As lentivirus, retrovirus and transgenes are integrated into the genome of a cell, their encoded miRNAs will likely follow the same biogenesis pathway and adopt the same mechanism of action of endogenous miRNAs. miRNA functions and mechanisms deduced from experiments employing these approaches will likely be physiologically relevant. Second, if transient transfection is the only method available to alter miRNA expression levels then miRNA mimics should be introduced into cells at transfection concentrations much lower than the 25 or 100 nM that are commonly used. Ideally, the cellular concentration of transfected miRNAs should be optimized by Northern blot and compared with that of primary cells in which these miRNAs are known to function. Supraphysiological expression levels and the appearance of high molecular weight RNA species, whose function is still unclear, should be avoided. Third, a control transfection with the same concentration of an unrelated miRNA mimic is essential for any miRNA mimic transfection experiments, as the transfection of miRNA mimics *per se* may alter gene expression in a non-specific manner. To reveal miRNA-specific effects, a seed-mutant form of the same miRNA mimic is highly recommended (Lim et al., 2005). Last, the translation repression followed by mRNA degradation model of miRNA mechanism of action should be interpreted with caution, as it remains to be investigated whether mammalian miRNAs expressed at physiological levels employ similar mechanisms of action (Jin and Xiao, 2015). Increasing numbers of mutant mouse strains harboring gain- and loss-of function mutations for individual miRNA genes are being generated (Olive et al., 2015). Primary cells from these animal models are ideal for studying miRNA mechanisms of action in the future.

During the revision of our manuscript, another cautionary tale on miRNA mimics was published, reporting that transient transfection of miRNA mimics may cause considerable side effects due to passenger strand (miRNA^*^) loading to AGO (Søkilde et al., 2015). Their study and our study investigated different aspects of miRNA mimics, and together provided a list of potential artifacts researchers should carefully consider, control, and avoid.

## Author contributions

AM conducted miR-155 transgenic mouse experiments under supervision of RR. ML produced control and miR-17~92-expressing lentiviruses. SK conducted human lymphoma cell line experiments under supervision of MM. SH provided technical support for small RNA deep sequencing experiments, while HJ and MS analyzed the deep sequencing data. HJ and AG designed and performed all other experiments. HJ and CX wrote the manuscript with contribution from all authors.

### Conflict of interest statement

The authors declare that the research was conducted in the absence of any commercial or financial relationships that could be construed as a potential conflict of interest.

## References

[B1] Agudo J. Ruzo A. Tung N. Salmon H. Leboeuf M. Hashimoto D.. (2014). The miR-126-VEGFR2 axis controls the innate response to pathogen-associated nucleic acids. Nat. Immunol. 15, 54–62. 10.1038/ni.2767 24270517 PMC3896265

[B2] Ambros V. (2004). The functions of animal microRNAs. Nature 431, 350–355. 10.1038/nature02871 15372042

[B3] Ameres S. L. Horwich M. D. Hung J. H. Xu J. Ghildiyal M. Weng Z.. (2010). Target RNA-directed trimming and tailing of small silencing RNAs. Science 328, 1534–1539. 10.1126/science.1187058 20558712 PMC2902985

[B4] Ameres S. L. Zamore P. D. (2013). Diversifying microRNA sequence and function. Nat. Rev. Mol. Cell Biol. 14, 475–488. 10.1038/nrm3611 23800994

[B5] Baek D. Villén J. Shin C. Camargo F. D. Gygi S. P. Bartel D. P. (2008). The impact of microRNAs on protein output. Nature 455, 64–71. 10.1038/nature07242 18668037 PMC2745094

[B6] Baumjohann D. Kageyama R. Clingan J. M. Morar M. M. Patel S. de Kouchkovsky D.. (2013). The microRNA cluster miR-17~92 promotes TFH cell differentiation and represses subset-inappropriate gene expression. Nat. Immunol. 14, 840–848. 10.1038/ni.2642 23812098 PMC3720769

[B7] Bazzini A. A. Lee M. T. Giraldez A. J. (2012). Ribosome profiling shows that miR-430 reduces translation before causing mRNA decay in zebrafish. Science 336, 233–237. 10.1126/science.1215704 22422859 PMC3547538

[B8] Bian S. Hong J. Li Q. Schebelle L. Pollock A. Knauss J. L.. (2013). MicroRNA cluster miR-17-92 regulates neural stem cell expansion and transition to intermediate progenitors in the developing mouse neocortex. Cell Rep. 3, 1398–1406. 10.1016/j.celrep.2013.03.037 23623502 PMC3762321

[B9] Biton M. Levin A. Slyper M. Alkalay I. Horwitz E. Mor H.. (2011). Epithelial microRNAs regulate gut mucosal immunity via epithelium-T cell crosstalk. Nat. Immunol. 12, 239–246. 10.1038/ni.1994 21278735

[B10] Boettger T. Beetz N. Kostin S. Schneider J. Krüger M. Hein L.. (2009). Acquisition of the contractile phenotype by murine arterial smooth muscle cells depends on the Mir143/145 gene cluster. J. Clin. Invest. 119, 2634–2647. 10.1172/JCI38864 19690389 PMC2735940

[B11] Boldin M. P. Taganov K. D. Rao D. S. Yang L. Zhao J. L. Kalwani M.. (2011). miR-146a is a significant brake on autoimmunity, myeloproliferation, and cancer in mice. J. Exp. Med. 208, 1189–1201. 10.1084/jem.20101823 21555486 PMC3173243

[B12] Bosson A. D. Zamudio J. R. Sharp P. A. (2014). Endogenous miRNA and target concentrations determine susceptibility to potential ceRNA competition. Mol. Cell 56, 347–359. 10.1016/j.molcel.2014.09.018 25449132 PMC5048918

[B13] Bushati N. Cohen S. M. (2007). microRNA functions. Annu. Rev. Cell Dev. Biol. 23, 175–205. 10.1146/annurev.cellbio.23.090506.123406 17506695

[B14] Calabrese J. M. Seila A. C. Yeo G. W. Sharp P. A. (2007). RNA sequence analysis defines Dicer's role in mouse embryonic stem cells. Proc. Natl. Acad. Sci. U.S.A. 104, 18097–18102. 10.1073/pnas.0709193104 17989215 PMC2084302

[B15] Callis T. E. Pandya K. Seok H. Y. Tang R. H. Tatsuguchi M. Huang Z. P.. (2009). MicroRNA-208a is a regulator of cardiac hypertrophy and conduction in mice. J. Clin. Invest. 119, 2772–2786. 10.1172/JCI36154 19726871 PMC2735902

[B16] Chen P. Y. Manninga H. Slanchev K. Chien M. C. Russo J. J. Ju J. Y.. (2005). The developmental miRNA profiles of zebrafish as determined by small RNA cloning. Genes Dev. 19, 1288–1293. 10.1101/gad.1310605 15937218 PMC1142552

[B17] Conkrite K. Sundby M. Mukai S. Thomson J. M. Mu D. Hammond S. M.. (2011). miR-17~92 cooperates with RB pathway mutations to promote retinoblastoma. Genes Dev. 25, 1734–1745. 10.1101/gad.17027411 21816922 PMC3165937

[B18] Costinean S. Zanesi N. Pekarsky Y. Tili E. Volinia S. Heerema N.. (2006). Pre-B cell proliferation and lymphoblastic leukemia/high-grade lymphoma in E(mu)-miR155 transgenic mice. Proc. Natl. Acad. Sci. U.S.A. 103, 7024–7029. 10.1073/pnas.0602266103 16641092 PMC1459012

[B19] Danielson L. S. Park D. S. Rotllan N. Chamorro-Jorganes A. Guijarro M. V. Fernandez-Hernando C.. (2013). Cardiovascular dysregulation of miR-17-92 causes a lethal hypertrophic cardiomyopathy and arrhythmogenesis. FASEB J. 27, 1460–1467. 10.1096/fj.12-221994 23271053 PMC3606524

[B20] De Pontual L. Yao E. Callier P. Faivre L. Drouin V. Cariou S.. (2011). Germline deletion of the miR-17 ~ 92 cluster causes skeletal and growth defects in humans. Nat. Genet. 43, 1026–U1146. 10.1038/ng.915 21892160 PMC3184212

[B21] Dewitte-Orr S. J. Mehta D. R. Collins S. E. Suthar M. S. Gale M. Jr. Mossman K. L. (2009). Long double-stranded RNA induces an antiviral response independent of IFN regulatory factor 3, IFN-beta promoter stimulator 1, and IFN. J. Immunol. 183, 6545–6553. 10.4049/jimmunol.0900867 19864603 PMC2885285

[B22] Dorsett Y. McBride K. M. Jankovic M. Gazumyan A. Thai T. H. Robbiani D. F.. (2008). MicroRNA-155 suppresses activation-induced cytidine deaminase-mediated Myc-Igh translocation. Immunity 28, 630–638. 10.1016/j.immuni.2008.04.002 18455451 PMC2713656

[B23] Du P. Wang L. Sliz P. Gregory R. I. (2015). A biogenesis step upstream of microprocessor controls miR-17~92 expression. Cell 162, 885–899. 10.1016/j.cell.2015.07.008 26255770 PMC4537828

[B24] Eis P. S. Tam W. Sun L. Chadburn A. Li Z. Gomez M. F.. (2005). Accumulation of miR-155 and BIC RNA in human B cell lymphomas. Proc. Natl. Acad. Sci. U.S.A. 102, 3627–3632. 10.1073/pnas.0500613102 15738415 PMC552785

[B25] Fabian M. R. Sonenberg N. Filipowicz W. (2010). Regulation of mRNA translation and stability by microRNAs. Annu. Rev. Biochem. 79, 351–379. 10.1146/annurev-biochem-060308-103103 20533884

[B26] Friedländer M. R. Chen W. Adamidi C. Maaskola J. Einspanier R. Knespel S.. (2008). Discovering microRNAs from deep sequencing data using miRDeep. Nat. Biotechnol. 26, 407–415. 10.1038/nbt1394 18392026

[B27] Garcia D. M. Baek D. Shin C. Bell G. W. Grimson A. Bartel D. P. (2011). Weak seed-pairing stability and high target-site abundance decrease the proficiency of lsy-6 and other microRNAs. Nat. Struct. Mol. Biol. 18, 1139–1146. 10.1038/nsmb.2115 21909094 PMC3190056

[B28] Giraldez A. J. Mishima Y. Rihel J. Grocock R. J. Van Dongen S. Inoue K.. (2006). Zebrafish MiR-430 promotes deadenylation and clearance of maternal mRNAs. Science 312, 75–79. 10.1126/science.1122689 16484454

[B29] Git A. (2012). Research tools: a recipe for disaster. Nature 484, 439–440. 10.1038/484439a 22538584

[B30] Grimson A. Farh K. K. H. Johnston W. K. Garrett-Engele P. Lim L. P. Bartel D. P. (2007). MicroRNA targeting specificity in mammals: determinants beyond seed pairing. Mol. Cell 27, 91–105. 10.1016/j.molcel.2007.06.017 17612493 PMC3800283

[B31] Guil S. Caceres J. F. (2007). The multifunctional RNA-binding protein hnRNP A1 is required for processing of miR-18a. Nat. Struct. Mol. Biol. 14, 591–596. 10.1038/nsmb1250 17558416

[B32] Guo H. Ingolia N. T. Weissman J. S. Bartel D. P. (2010). Mammalian microRNAs predominantly act to decrease target mRNA levels. Nature 466, 835–840. 10.1038/nature09267 20703300 PMC2990499

[B33] Ha M. Kim V. N. (2014). Regulation of microRNA biogenesis. Nat. Rev. Mol. Cell Biol. 15, 509–524. 10.1038/nrm3838 25027649

[B34] Hasuwa H. Ueda J. Ikawa M. Okabe M. (2013). MiR-200b and miR-429 function in mouse ovulation and are essential for female fertility. Science 341, 71–73. 10.1126/science.1237999 23765281

[B35] He L. Thomson J. M. Hemann M. T. Hernando-Monge E. Mu D. Goodson S.. (2005). A microRNA polycistron as a potential human oncogene. Nature 435, 828–833. 10.1038/nature03552 15944707 PMC4599349

[B36] Henao-Mejia J. Williams A. Goff L. A. Staron M. Licona-Limón P. Kaech S. M.. (2013). The microRNA miR-181 is a critical cellular metabolic rheostat essential for NKT cell ontogenesis and lymphocyte development and homeostasis. Immunity 38, 984–997. 10.1016/j.immuni.2013.02.021 23623381 PMC3738211

[B37] Hong L. X. Lai M. Y. Chen M. Xie C. C. Liao R. Kang Y. J.. (2010). The miR-17-92 cluster of MicroRNAs confers tumorigenicity by inhibiting oncogene-induced senescence. Cancer Res. 70, 8547–8557. 10.1158/0008-5472.CAN-10-1938 20851997 PMC2970743

[B38] Jacobson B. S. Ryan U. S. (1982). Growth of endothelial and hela-cells on a new multipurpose microcarrier that is positive, negative or collagen coated. Tissue Cell 14, 69–83. 10.1016/0040-8166(82)90008-8 7089966

[B39] Janas M. M. Wang B. B. Harris A. S. Aguiar M. Shaffer J. M. Subrahmanyam Y. V. B. K.. (2012). Alternative RISC assembly: binding and repression of microRNA-mRNA duplexes by human Ago proteins. RNA 18, 2041–2055. 10.1261/rna.035675.112 23019594 PMC3479394

[B40] Jiang S. Li C. R. Olive V. Lykken E. Feng F. Sevilla J.. (2011). Molecular dissection of the miR-17-92 cluster's critical dual roles in promoting Th1 responses and preventing inducible Treg differentiation. Blood 118, 5487–5497. 10.1182/blood-2011-05-355644 21972292 PMC3217351

[B41] Jin H. Y. Lai M. Y. Xiao C. C. (2014). microRNA-17~92 is a powerful cancer driver and a therapeutic target. Cell Cycle (Georgetown, Tex) 13, 495–496. 10.4161/cc.27784 24419145 PMC6317711

[B42] Jin H. Y. Oda H. Lai M. Skalsky R. L. Bethel K. Shepherd J.. (2013). MicroRNA-17~92 plays a causative role in lymphomagenesis by coordinating multiple oncogenic pathways. EMBO J. 32, 2377–2391. 10.1038/emboj.2013.178 23921550 PMC3771343

[B43] Jin H. Y. Xiao C. (2015). MicroRNA mechanisms of action: what have we learned from mice? Front. Genet. 6:328 10.3389/fgene.2015.00328 PMC4644800 26635864

[B44] Jonas S. Izaurralde E. (2015). Towards a molecular understanding of microRNA-mediated gene silencing. Nat. Rev. Genet. 16, 421–433. 10.1038/nrg3965 26077373

[B45] Kang S. G. Liu W. H. Lu P. Jin H. Y. Lim H. W. Shepherd J.. (2013). MicroRNAs of the miR-17~92 family are critical regulators of T(FH) differentiation. Nat. Immunol. 14, 849–857. 10.1038/ni.2648 23812097 PMC3740954

[B46] Khan A. A. Penny L. A. Yuzefpolskiy Y. Sarkar S. Kalia V. (2013). MicroRNA-17~92 regulates effector and memory CD8 T-cell fates by modulating proliferation in response to infections. Blood 121, 4473–4483. 10.1182/blood-2012-06-435412 23596046

[B47] Kim V. N. Han J. Siomi M. C. (2009). Biogenesis of small RNAs in animals. Nat. Rev. Mol. Cell Biol. 10, 126–139. 10.1038/nrm2632 19165215

[B48] Krol J. Loedige I. Filipowicz W. (2010). The widespread regulation of microRNA biogenesis, function and decay. Nat. Rev. Genet. 11, 597–610. 10.1038/nrg2843 20661255

[B49] Kuchen S. Resch W. Yamane A. Kuo N. Li Z. Chakraborty T.. (2010). Regulation of microRNA expression and abundance during lymphopoiesis. Immunity 32, 828–839. 10.1016/j.immuni.2010.05.009 20605486 PMC2909788

[B50] Larsson O. Nadon R. (2013). Re-analysis of genome wide data on mammalian microRNA-mediated suppression of gene expression. Translation 1:e24557 10.4161/trla.24557 PMC4718065 26824020

[B51] Lee R. C. Feinbaum R. L. Ambros V. (1993). The *C. elegans* heterochronic gene lin-4 encodes small RNAs with antisense complementarity to lin-14. Cell 75, 843–854. 10.1016/0092-8674(93)90529-Y 8252621

[B52] Lim L. P. Lau N. C. Garrett-Engele P. Grimson A. Schelter J. M. Castle J.. (2005). Microarray analysis shows that some microRNAs downregulate large numbers of target mRNAs. Nature 433, 769–773. 10.1038/nature03315 15685193

[B53] Liu N. Bezprozvannaya S. Shelton J. M. Frisard M. I. Hulver M. W. McMillan R. P.. (2011). Mice lacking microRNA 133a develop dynamin 2-dependent centronuclear myopathy. J. Clin. Invest. 121, 3258–3268. 10.1172/JCI46267 21737882 PMC3148737

[B54] Lu J. Getz G. Miska E. A. Alvarez-Saavedra E. Lamb J. Peck D.. (2005). MicroRNA expression profiles classify human cancers. Nature 435, 834–838. 10.1038/nature03702 15944708

[B55] Lu L. F. Thai T. H. Calado D. P. Chaudhry A. Kubo M. Tanaka K.. (2009). Foxp3-dependent microRNA155 confers competitive fitness to regulatory T cells by targeting SOCS1 protein. Immunity 30, 80–91. 10.1016/j.immuni.2008.11.010 19144316 PMC2654249

[B56] Lu Y. Thomson J. M. Wong H. Y. Hammond S. M. Hogan B. L. (2007). Transgenic over-expression of the microRNA miR-17-92 cluster promotes proliferation and inhibits differentiation of lung epithelial progenitor cells. Dev. Biol. 310, 442–453. 10.1016/j.ydbio.2007.08.007 17765889 PMC2052923

[B57] Ma X. D. Kumar M. Choudhury S. N. Buscaglia L. E. B. Barker J. R. Kanakamedala K.. (2011). Loss of the miR-21 allele elevates the expression of its target genes and reduces tumorigenesis. Proc. Natl. Acad. Sci. U.S.A. 108, 10144–10149. 10.1073/pnas.1103735108 21646541 PMC3121848

[B58] Martin M. (2011). Cutadapt removes adapter sequences from high-throughput sequencing reads. EMBnet. J. 17, 10–12. 10.14806/ej.17.1.200

[B59] Morgan M. Anders S. Lawrence M. Aboyoun P. Pagés H. Gentleman R. (2009). ShortRead: a bioconductor package for input, quality assessment and exploration of high-throughput sequence data. Bioinformatics 25, 2607–2608. 10.1093/bioinformatics/btp450 19654119 PMC2752612

[B60] Mukherji S. Ebert M. S. Zheng G. X. Tsang J. S. Sharp P. A. Van Oudenaarden A. (2011). MicroRNAs can generate thresholds in target gene expression. Nat. Genet. 43, 854–859. 10.1038/ng.905 21857679 PMC3163764

[B61] Neilson J. R. Zheng G. X. Y. Burge C. B. Sharp P. A. (2007). Dynamic regulation of miRNA expression in ordered stages of cellular development. Genes Dev. 21, 578–589. 10.1101/gad.1522907 17344418 PMC1820899

[B62] O'connell R. M. Chaudhuri A. A. Rao D. S. Baltimore D. (2009). Inositol phosphatase SHIP1 is a primary target of miR-155. Proc. Natl. Acad. Sci. U.S.A. 106, 7113–7118. 10.1073/pnas.0902636106 19359473 PMC2678424

[B63] O'connell R. M. Kahn D. Gibson W. S. Round J. L. Scholz R. L. Chaudhuri A. A.. (2010). MicroRNA-155 promotes autoimmune inflammation by enhancing inflammatory T cell development. Immunity 33, 607–619. 10.1016/j.immuni.2010.09.009 20888269 PMC2966521

[B64] Olive V. Minella A. C. He L. (2015). Outside the coding genome, mammalian microRNAs confer structural and functional complexity. Sci. Signal. 8, re2. 10.1126/scisignal.2005813 25783159 PMC4425368

[B65] Palanichamy J. K. Rao D. S. (2014). miRNA dysregulation in cancer: towards a mechanistic understanding. Front. Genet. 5:54. 10.3389/fgene.2014.00054 24672539 PMC3957189

[B66] Pedersen I. M. Otero D. Kao E. Miletic A. V. Hother C. Ralfkiaer E.. (2009). Onco-miR-155 targets SHIP1 to promote TNFalpha-dependent growth of B cell lymphomas. EMBO Mol. Med. 1, 288–295. 10.1002/emmm.200900028 19890474 PMC2771872

[B67] Rai D. Karanti S. Jung I. Dahia P. L. Aguiar R. C. (2008). Coordinated expression of microRNA-155 and predicted target genes in diffuse large B-cell lymphoma. Cancer Genet. Cytogenet. 181, 8–15. 10.1016/j.cancergencyto.2007.10.008 18262046 PMC2276854

[B68] Rao E. Jiang C. Ji M. Huang X. Iqbal J. Lenz G.. (2012). The miRNA-17~92 cluster mediates chemoresistance and enhances tumor growth in mantle cell lymphoma via PI3K/AKT pathway activation. Leukemia 26, 1064–1072. 10.1038/leu.2011.305 22116552

[B69] Reynolds A. Anderson E. M. Vermeulen A. Fedorov Y. Robinson K. Leake D.. (2006). Induction of the interferon response by siRNA is cell type- and duplex length-dependent. RNA 12, 988–993. 10.1261/rna.2340906 16611941 PMC1464853

[B70] Rickert R. C. Roes J. Rajewsky K. (1997). B lymphocyte-specific, Cre-mediated mutagenesis in mice. Nucleic Acids Res. 25, 1317–1318. 10.1093/nar/25.6.1317 9092650 PMC146582

[B71] Rodriguez A. Vigorito E. Clare S. Warren M. V. Couttet P. Soond D. R.. (2007). Requirement of bic/microRNA-155 for normal immune function. Science 316, 608–611. 10.1126/science.1139253 17463290 PMC2610435

[B72] Sanuki R. Onishi A. Koike C. Muramatsu R. Watanabe S. Muranishi Y.. (2011). miR-124a is required for hippocampal axogenesis and retinal cone survival through Lhx2 suppression. Nat. Neurosci. 14, 1125–1134. 10.1038/nn.2897 21857657

[B73] Schmitz R. Young R. M. Ceribelli M. Jhavar S. Xiao W. Zhang M.. (2012). Burkitt lymphoma pathogenesis and therapeutic targets from structural and functional genomics. Nature 490, 116–120. 10.1038/nature11378 22885699 PMC3609867

[B74] Selbach M. Schwanhäusser B. Thierfelder N. Fang Z. Khanin R. Rajewsky N. (2008). Widespread changes in protein synthesis induced by microRNAs. Nature 455, 58–63. 10.1038/nature07228 18668040

[B75] Shibata M. Nakao H. Kiyonari H. Abe T. Aizawa S. (2011). MicroRNA-9 regulates neurogenesis in mouse telencephalon by targeting multiple transcription factors. J. Neurosci. 31, 3407–3422. 10.1523/JNEUROSCI.5085-10.2011 21368052 PMC6623912

[B76] Søkilde R. Newie I. Persson H. Borg Å. Rovira C. (2015). Passenger strand loading in overexpression experiments using microRNA mimics. RNA Biol. 12, 787–791. 10.1080/15476286.2015.1020270 26121563 PMC4615182

[B77] Stadthagen G. Tehler D. Høyland-Kroghsbo N. M. Wen J. Krogh A. Jensen K. T.. (2013). Loss of miR-10a activates lpo and collaborates with activated Wnt signaling in inducing intestinal neoplasia in female mice. PLoS Genet. 9:e1003913. 10.1371/journal.pgen.1003913 24204315 PMC3812087

[B78] Thai T. H. Calado D. P. Casola S. Ansel K. M. Xiao C. Xue Y.. (2007). Regulation of the germinal center response by microRNA-155. Science 316, 604–608. 10.1126/science.1141229 17463289

[B79] Thatcher E. J. Bond J. Paydar I. Patton J. G. (2008). Genomic organization of zebrafish microRNAs. BMC Genomics 9:253. 10.1186/1471-2164-9-253 18510755 PMC2427041

[B80] Thomson D. W. Bracken C. P. Szubert J. M. Goodall G. J. (2013). On measuring miRNAs after transient transfection of mimics or antisense inhibitors. PLoS ONE 8:e55214. 10.1371/journal.pone.0055214 23358900 PMC3554668

[B81] Van Rooij E. Sutherland L. B. Qi X. Richardson J. A. Hill J. Olson E. N. (2007). Control of stress-dependent cardiac growth and gene expression by a microRNA. Science 316, 575–579. 10.1126/science.1139089 17379774

[B82] Ventura A. Young A. G. Winslow M. M. Lintault L. Meissner A. Erkeland S. J.. (2008). Targeted deletion reveals essential and overlapping functions of the miR-17~92 family of miRNA clusters. Cell 132, 875–886. 10.1016/j.cell.2008.02.019 18329372 PMC2323338

[B83] Vigorito E. Perks K. L. Abreu-Goodger C. Bunting S. Xiang Z. Kohlhaas S.. (2007). microRNA-155 regulates the generation of immunoglobulin class-switched plasma cells. Immunity 27, 847–859. 10.1016/j.immuni.2007.10.009 18055230 PMC4135426

[B84] Wang D. Zhang Z. O'loughlin E. Wang L. Fan X. Lai E. C.. (2013). MicroRNA-205 controls neonatal expansion of skin stem cells by modulating the PI(3)K pathway. Nat. Cell Biol. 15, 1153–1163. 10.1038/ncb2827 23974039 PMC3789848

[B85] Wang K. Liu C. Y. Zhang X. J. Feng C. Zhou L. Y. Zhao Y.. (2015). miR-361-regulated prohibitin inhibits mitochondrial fission and apoptosis and protects heart from ischemia injury. Cell Death Differ. 22, 1058–1068. 10.1038/cdd.2014.200 25501599 PMC4423187

[B86] Wang Q. Carmichael G. G. (2004). Effects of length and location on the cellular response to double-stranded RNA. Microbiol. Mol. Biol. Rev. 68, 432–452. 10.1128/MMBR.68.3.432-452.2004 15353564 PMC515255

[B87] Wang Z. (2011). The guideline of the design and validation of MiRNA mimics. Methods Mol. Biol. 676, 211–223. 10.1007/978-1-60761-863-8_15 20931400

[B88] Wightman B. Ha I. Ruvkun G. (1993). Posttranscriptional regulation of the heterochronic gene lin-14 by lin-4 mediates temporal pattern formation in *C. elegans*. Cell 75, 855–862. 10.1016/0092-8674(93)90530-4 8252622

[B89] Wilczynska A. Bushell M. (2015). The complexity of miRNA-mediated repression. Cell Death Differ. 22, 22–33. 10.1038/cdd.2014.112 25190144 PMC4262769

[B90] Williams A. H. Valdez G. Moresi V. Qi X. McAnally J. Elliott J. L.. (2009). MicroRNA-206 delays ALS progression and promotes regeneration of neuromuscular synapses in mice. Science 326, 1549–1554. 10.1126/science.1181046 20007902 PMC2796560

[B91] Xiao C. Calado D. P. Galler G. Thai T. H. Patterson H. C. Wang J.. (2007). MiR-150 controls B cell differentiation by targeting the transcription factor c-Myb. Cell 131, 146–159. 10.1016/j.cell.2007.07.021 17923094

[B92] Xiao C. Srinivasan L. Calado D. P. Patterson H. C. Zhang B. Wang J.. (2008). Lymphoproliferative disease and autoimmunity in mice with increased miR-17-92 expression in lymphocytes. Nat. Immunol. 9, 405–414. 10.1038/ni1575 18327259 PMC2533767

[B93] Xu S. Ou X. Huo J. Lim K. Huang Y. Chee S.. (2015). Mir-17-92 regulates bone marrow homing of plasma cells and production of immunoglobulin G2c. Nat. Commun. 6, 6764. 10.1038/ncomms7764 25881561

[B94] Zhao Y. Ransom J. F. Li A. Vedantham V. Von Drehle M. Muth A. N.. (2007). Dysregulation of cardiogenesis, cardiac conduction, and cell cycle in mice lacking miRNA-1-2. Cell 129, 303–317. 10.1016/j.cell.2007.03.030 17397913

[B95] Zhao Y. Samal E. Srivastava D. (2005). Serum response factor regulates a muscle-specific microRNA that targets Hand2 during cardiogenesis. Nature 436, 214–220. 10.1038/nature03817 15951802

